# Alloying Germanium Nanowire Anodes Dramatically Outperform Graphite Anodes in Full-Cell Chemistries over a Wide Temperature Range

**DOI:** 10.1021/acsaem.0c02928

**Published:** 2021-02-02

**Authors:** Gearoid A. Collins, Karrina McNamara, Seamus Kilian, Hugh Geaney, Kevin M. Ryan

**Affiliations:** † Department of Chemical Sciences and Bernal Institute, University of Limerick, Limerick V94 T9PX, Ireland; ‡ Department of Physics, University of Limerick, Limerick V94 T9PX, Ireland

**Keywords:** germanium nanowire, graphite, lithium-ion battery, full cell, wide temperature performance, temperature-controlled electrochemical amorphization

## Abstract

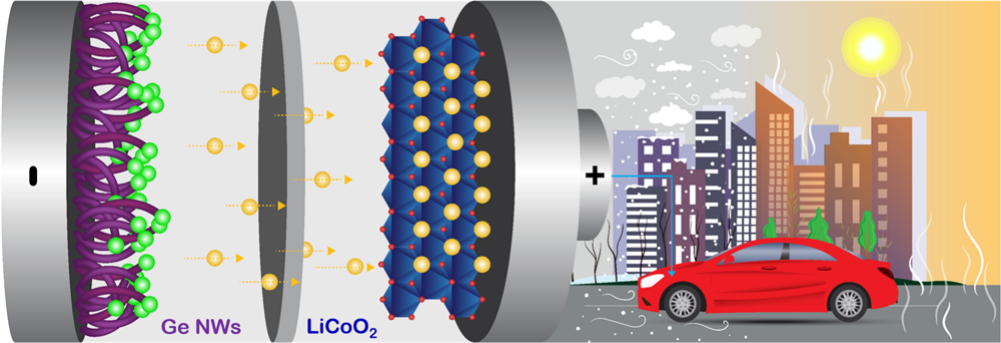

The electrochemical performance of Ge, an alloying anode in the form of directly grown nanowires (NWs), in Li-ion full cells (vs LiCoO_2_) was analyzed over a wide temperature range (−40 to 40 °C). LiCoO_2_||Ge cells in a standard electrolyte exhibited specific capacities 30× and 50× those of LiCoO_2_||C cells at −20 and −40 °C, respectively. We further show that propylene carbonate addition further improved the low-temperature performance of LiCoO_2_||Ge cells, achieving a specific capacity of 1091 mA h g^–1^ after 400 cycles when charged/discharged at −20 °C. At 40 °C, an additive mixture of ethyl methyl carbonate and lithium bis(oxalato)borate stabilized the capacity fade from 0.22 to 0.07% cycle^–1^. Similar electrolyte additives in LiCoO_2_||C cells did not allow for any gains in performance. Interestingly, the capacity retention of LiCoO_2_||Ge improved at low temperatures due to delayed amorphization of crystalline NWs, suppressing complete lithiation and high-order Li_15_Ge_4_ phase formation. The results show that alloying anodes in suitably configured electrolytes can deliver high performance at the extremes of temperature ranges where electric vehicles operate, conditions that are currently not viable for commercial batteries without energy-inefficient temperature regulation.

## Introduction

The rate of displacement of conventional internal combustion engine (ICE) vehicles by electric vehicles (EVs) is strongly dependent on key performance gains in Li-ion batteries (LIBs), particularly regarding the range and fast charging. However, the adaptability of Li-ion cells to different climates in which ICE vehicles can operate is still a major issue that has yet to be adequately addressed. Typically, 18,650 cells operate well between 0 and 30 °C,^1,2^ but variations in cell temperature outside of this range trigger rapid capacity fade or a significant reduction in overall performance. The diminished performance of EVs in hot climates is also a key issue, with high-temperature cycling of the battery inducing rapid capacity fade and an overall reduction in power output.^3^ Similarly, EVs operating in cold climates experience a diminished range due to a combination of insufficient charging and a significant amount of power being redirected to heat the vehicle.^4^ The inability of EVs to operate under cold conditions also has important environmental implications considering that cold starts of ICE vehicles dramatically reduce fuel efficiency, leading to greater emissions.^5,6^ There is also a wider interest in enhancing the low-temperature performance of Li-ion cells for aerospace applications that require high storage capabilities over an extended period under severe environmental conditions.^7,8^ Extending the operable temperature range for Li-ion cells is thus of huge significance at high and low extremes.

The high-temperature performance of LIBs is limited by the instability of the liquid electrolyte and its susceptibility to degradation.^9^ The solid electrolyte interphase (SEI), formed during first cycle lithiation, plays a critical role in cell stability, removing direct contact at the electrode–electrolyte interface. Under normal conditions, electrolyte decomposition of the LiPF_6_ salt is a simple dissociation process, forming LiF and PF_5_, both of which aid SEI formation.^10^ However, high-temperature cycling promotes increased HF formation along with the buildup of internal pressure through gas evolution.^11−13^ High-temperature additives such as lithium bis(oxalato)borate (LiBOB) and ethyl methyl carbonate (EMC) have appeared as viable additives to suppress electrolyte decomposition reactions and in turn improve overall cell stability.^14−16^

Low temperatures dramatically increase cell impedance, increasing SEI layer resistance and charge transfer resistance (*R*_CT_) and suppressing Li ion transport through the cell. A combination of poor lithium diffusivity through the SEI layer and low conductivity of the graphite anode leads to a substantial drop in power output, as the LIB cells cannot sufficiently charge/discharge.^17−20^ Commercial cells lose the majority of their capacity below −10 °C, with 18,650 cells returning around 50% of their room temperature capacity when discharged at −20 °C, with a substantial drop to 5% below −40 °C.^18^ At low temperatures, cell output becomes strongly dependent on applied current density. At −10 °C, 18,650 cells lose the entirety of their energy when discharged at 1.5C.^21^ Electrochemical impedance spectroscopy (EIS) of graphite cells reveals that at low temperatures, cell impedance is dominated by a substantial increase in *R*_CT_.^22^ Consequently, the electrical characteristics of the anode material are critical in dictating the overall cell performance.

Recently, Li-ion alloying anodes in the form of group-IV Si and Ge materials have appeared as candidates to replace graphite intercalation anodes, owing to their high gravimetric capacities and high volumetric energy densities.^23−30^ Nanoscaling of the anode allows a significant increase in electronic and ionic conductivities, facilitating fast charging by increasing the electrode–electrolyte contact area and shortening the diffusion length for Li-ion transport.^31,32^ Moreover, shortened diffusion lengths and higher surface area/volume ratios can better overcome reduced conductivity at low temperatures. Ge nanowires (NWs) have been routinely shown to offer stable, high capacity cycling with a great propensity for high current densities.^33−35^ In this study, we show that an alloying anode (Ge NW-based) substantially outperforms conventional graphite anodes at high and low temperatures in full-cell configurations using “standard” electrolytes. Significant performance enhancements beyond this benchmark were obtained through careful electrolyte modifications, with the enhanced performance explained through ex situ analysis of morphological and SEI compositional changes. A comparison table to similar published work is provided in Table S1.

## Results and Discussion

**Figure 1:**
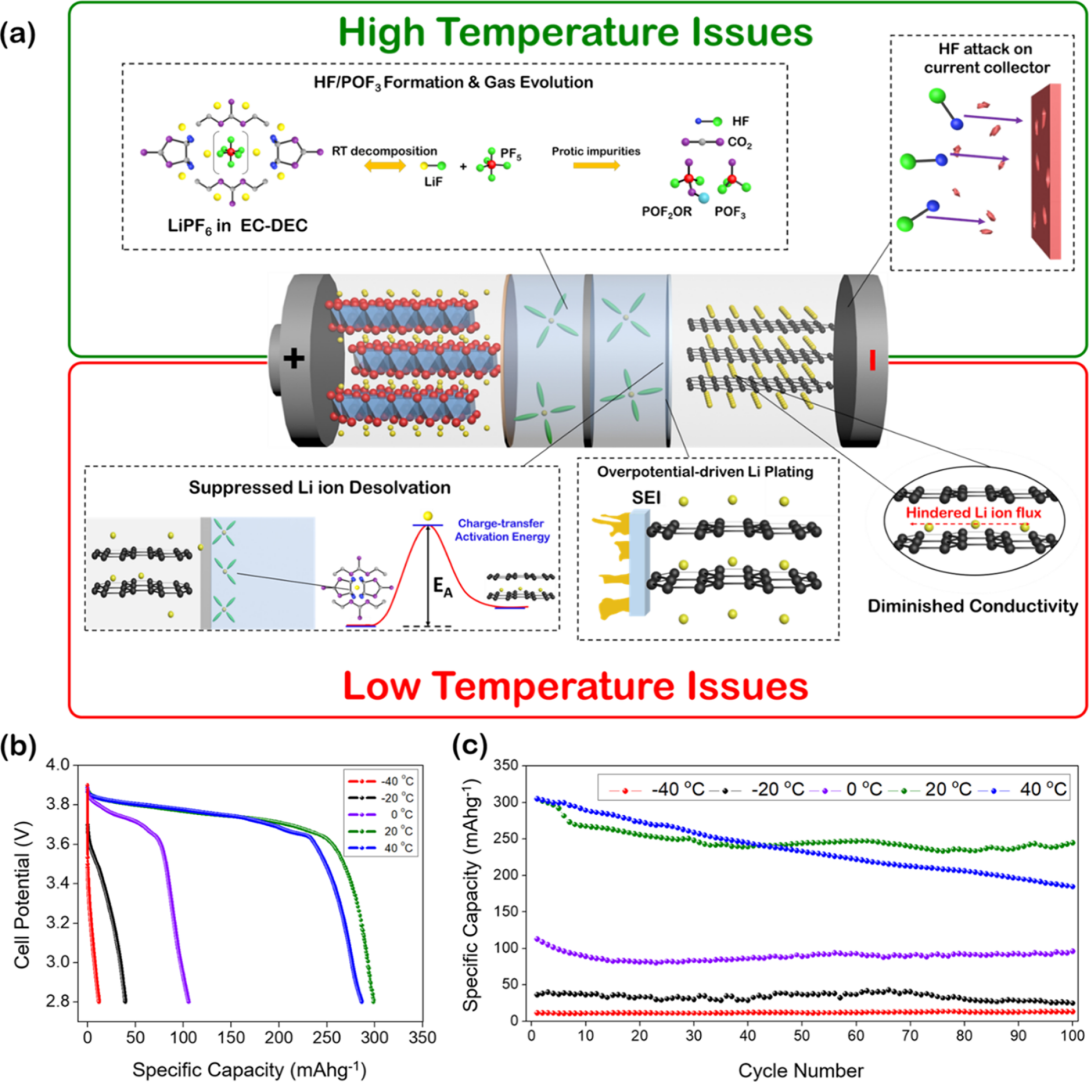
(a) Schematic illustration of the high- and low-temperature issues of conventional graphite-based cells. (b) Discharge capacity profiles (C/2 rate) and (c) discharge capacities (at 1C) of LiCoO_2_||C cells from −40 to 40 °C, charged and discharged symmetrically at the same temperature. Cells were cycled over a potential window of 2.8–3.9 V. The standard 1 M LiPF_6_ in EC–DEC (1:1 v/v) + 3 wt % VC was chosen for testing and kept constant between tests.

High- and low-temperature cycling of conventional graphite-based cells impose dramatic consequences on capacity output, cell stability, and rate capability. Figure 1a summarizes the major anode-specific issues of adverse temperatures on the cyclability of a LiCoO_2_||C cell. At low temperatures, graphite intercalation is predicated by huge increases in *R*_CT_ as low temperatures suppress Li-ion desolvation at the interface and diffusion through the graphite layers and exacerbate the propensity for Li plating through electrode polarization and anodic overcharging.^36−38^ At elevated temperatures, the instability of the conventional LiPF_6_ electrolyte leads to HF/POF_3_ formation along with CO_2_ evolution.^2,39^ Subsequent HF etching of the electrode current collectors manifests as irreversible capacity loss and eventual cell failure.^40,41^ The performance impacts of these established temperature-specific issues were analyzed through galvanostatic cycling of LiCoO_2_||C cells between the temperature limits of −40 and 40 °C (Figure 1b,c). A 1 M LiPF_6_ in EC–DEC (1:1 v/v) + 3 wt % VC was chosen as the electrolyte and denoted as the “standard” or “std.” for all subsequent testing. From the discharge capacity profiles in Figure 1b, LiCoO_2_||C returned a capacity of 301 mA h g^–1^ @ 20 °C, dropping to 106 mA h g^–1^ @ 0 °C and to 39 mA h g^–1^ @ –20 °C, and finally tapering to a negligible 11 mA h g^–1^ @ –40 °C. Discharge capacity aging tests of LiCoO_2_||C between these limits highlighted the strong dependence of LiCoO_2_||C cell performance on cell temperature (Figure 1c). At 40 °C, LiCoO_2_||C exhibited rapid capacity fade, with the initial 306 mA h g^–1^ falling to 185 mA h g^–1^ after 100 cycles. At low temperatures, the initial low capacity does not significantly change, suggesting a continuous inability to charge/discharge at 1C. For a 1C rate, the applied current is calculated to set the charge/discharge time to be 1 h, based on the theoretical capacity of Ge. From the published work, a value of 1384 mA h g^–1^ is widely acknowledged as the capacity of Ge.^33,42−45^ Poor capacity retention at elevated temperatures and low capacity at low temperatures reflect the respective issues of electrolyte instability and hindered Li-ion diffusion kinetics.

Comparative testing of LiCoO_2_||Ge cells revealed a far superior ability of Ge NWs to adapt to temperature variations. Preliminary testing of LiCoO_2_||Ge with PN ratios ranging from PN1–PN2.5 revealed PN1.5 cells as the best-performing cell configuration, maximizing specific energy and power and eliminating capacity limitations of Li-deficient PN1 cells (Figure S1). As a result, PN1.5 cells were chosen as the optimal cell configuration for testing. Furthermore, performance variations from increasing the PN ratio can be attributed to two major factors: (i) excess Li to maximize anode capacity and (ii) reduced anode loading. To analyze the effect of anode loading over the PN1–PN2.5 loading range, LiCoO_2_||Ge cells of equivalent PN ratios (PN1.5) were compared with low (0.25 mg cm^–2^)- and high (0.63 mg cm^–2^)-loading anodes (Figure S2). Setting a constant PN1.5 isolated loading-driven electrochemical performance variations (Figure S2). Overall, both cell loadings (0.25 and 0.63 mg cm^2^) exhibited similar capacity trends, reaching 91.61 and 91.42% capacity retention after 100 cycles (Figure S2a). Low-loading LiCoO_2_||Ge delivered a slightly higher average specific capacity than high-loading LiCoO_2_||Ge, which is attributed to a shorter anode diffusion length. Over the entire anode loading range of 0.25–0.63 mg cm^2^, higher loadings did not largely affect cell performance, exhibiting similar capacity retention and an average reduction in the specific capacity of 7.1% for 1C rate. Consequently, significant performance variations of different PN ratio cells in Figure S1a (47.6% capacity loss from PN2.5 and PN1 for 1C) can therefore be largely attributed to insufficient Li of PN1 cells, leading to incomplete anode lithiation and a subsequent reduction in specific capacity, energy, and power. Insufficient Li of PN1 cells is a well understood problem and current prelithiation studies may offer a future solution to this capacity balancing issue.^45−47^

**Figure 2:**
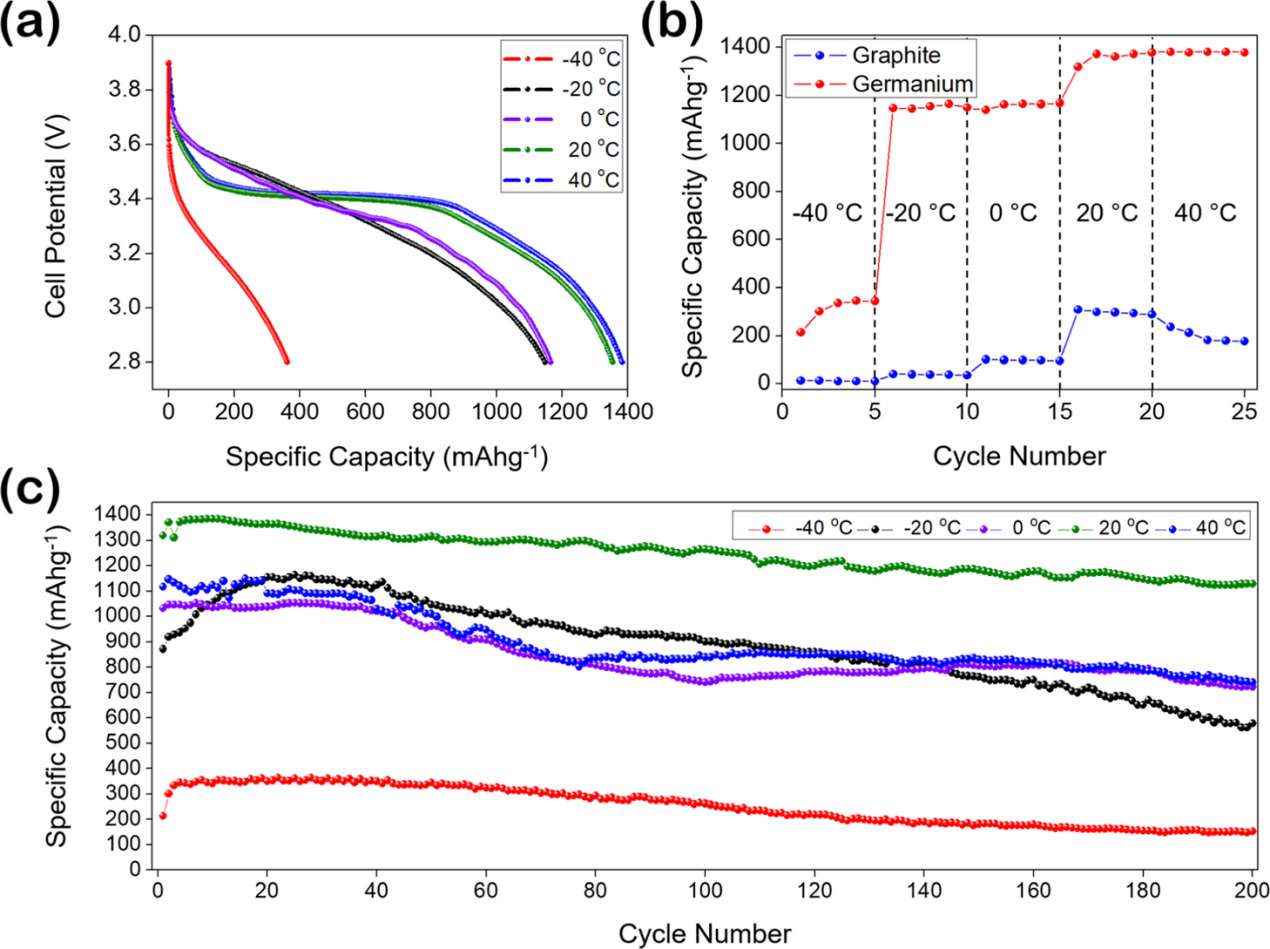
(a) Discharge capacity profiles for LiCoO_2_||Ge cells from −40 to 40 °C cycled at C/2. (b) “Temperature capability” testing of a LiCoO_2_||C (blue) and LiCoO_2_||Ge (red) cell from −40 to 40 °C at C/2 and (c) discharge capacities of LiCoO_2_||Ge cells cycled from −40 to 40 °C at a 1C rate. The standard 1 M LiPF_6_ in EC–DEC (1:1 v/v) + 3 wt % VC was chosen for testing and kept constant between tests.

Employing the std. electrolyte and 1C rate, the LiCoO_2_||Ge cell reached 1379 mA h g^–1^ at 40 °C and 1351 mA h g^–1^ @ 20 °C, dropping to 1164 mA h g^–1^@0 °C (84.1% of *Q*_th-Ge_) and then reducing marginally to 1143 mA h g^–1^@–20 °C (Figure 2a). As the cell temperature was lowered further to −40 °C, the cell capacity fell to 362 mA h g^–1^ (26.2% of *Q*_th-Ge_). Despite this drop-off in capacity @–40 °C, LiCoO_2_||Ge reaches 97.3% of the theoretical capacity of graphite. Differential capacity plots (DCPs) of the first-cycle lithiation/delithiation of LiCoO_2_||Ge from −40 to 40 °C are outlined in Figure S3. “Temperature capability” testing (an analogue of commonly used rate capability) was carried out on both cell types to compare the response of both anode types to variant temperatures (Figure 2b). Cells were cycled for five cycles at a C/2 rate, beginning at −40 °C, and the temperature was increased in 20 °C increments after every fifth cycle. The results represent the response of the two material types to variations in cell temperature. For both cell types, reduced capacity at low temperatures is shown to be reversible, with both cells reaching close to their theoretical capacity when cycled at 20 °C (98.1% of *Q*_th-Ge_ and 82.9% of *Q*_th-C_). The LiCoO_2_||Ge cell retains high capacity at temperatures as low as −20 °C (83.5% of *Q*_th-Ge_), whereas the LiCoO_2_||C cell returns an almost negligible capacity at this temperature. The LiCoO_2_||Ge cell exhibited an experimental capacity 30-fold that of LiCoO_2_||C at −20 °C and 40-fold at −40 °C. Between the temperature constraints of −20 to 40 °C, LiCoO_2_||Ge cells offer high capacity cycling, largely unaffected by variations in cell temperature. In contrast, the performance of LiCoO_2_||C cells was strongly dictated by cell temperature. Corresponding specific energy and power densities of these cells as a function of temperature are outlined in Figure S4.

A combination of cross-sectional scanning electron microscopy (SEM) and EIS correlated performance differences between LiCoO_2_||C and LiCoO_2_||Ge cells to *R*_CT_ variations and the corresponding electrode layer thicknesses of the anodes (Figure S5). The diminished capacity of graphite at low temperatures is governed by a huge increase in *R*_CT_, suppressing interfacial Li-ion desolvation. LiCoO_2_||Ge exhibits a room temperature *R*_CT_ of 43 Ω, rising to 1.1 × 10^3^ Ω at −40 °C (Figure S5c,d). Comparatively, the *R*_CT_ of the LiCoO_2_||C cell at 20 °C was found to be 682 Ω, rising to 3 × 10^5^ Ω at −40 °C (Figure S5a,b). For Ge and graphite cells of equivalent areal capacities, LiCoO_2_||C cells exhibit a *R*_CT_ 27× that of LiCoO_2_||Ge at −40 °C. The extremely high *R*_CT_ of LiCoO_2_||C cells at −40 °C explains their inability to charge/discharge at low temperatures. Cross-sectional SEM was carried out on graphite and Ge anodes. Both anodes were capacity-matched areally with respective mass loadings of 1.74 and 0.42 mg cm^–2^ (*Q*_th_ = 372 μA h electrode^–1^). SEM of the pristine anodes found that the graphite and Ge anodes have active layer thicknesses of 102 and 12 μm, respectively (Figure S5e,f). Overall, for capacity-matched anodes, the graphite layer has a thickness 8.5× that of Ge, leading to higher cell impedance associated with longer Li-ion diffusion lengths and lower surface area/volume ratios. The RT rate performance of LiCoO_2_||Ge and LiCoO_2_||C cells of equivalent areal capacities was tested and cells were compared in terms of specific capacity, energy, and power (Figure S6). Overall, LiCoO_2_||Ge delivered far superior energy density for every current density. Such disparity in performance is further heightened at low temperatures. The long-term stability of LiCoO_2_||Ge cells, employing the std. electrolyte configuration, at different temperatures was tested (Figure 2c). At 40 °C, LiCoO_2_||Ge exhibited rapid capacity fade, similar to LiCoO_2_||C, retaining 65.77% capacity after 200 cycles. Similarly, poor capacity retentions were noted at −40, −20, and 0 °C, retaining 44.09, 48.41, and 68.57%, respectively. Despite the high initial capacities at low temperatures, LiCoO_2_||Ge is limited by the inefficiencies of the std. electrolyte configuration.

**Figure 3:**
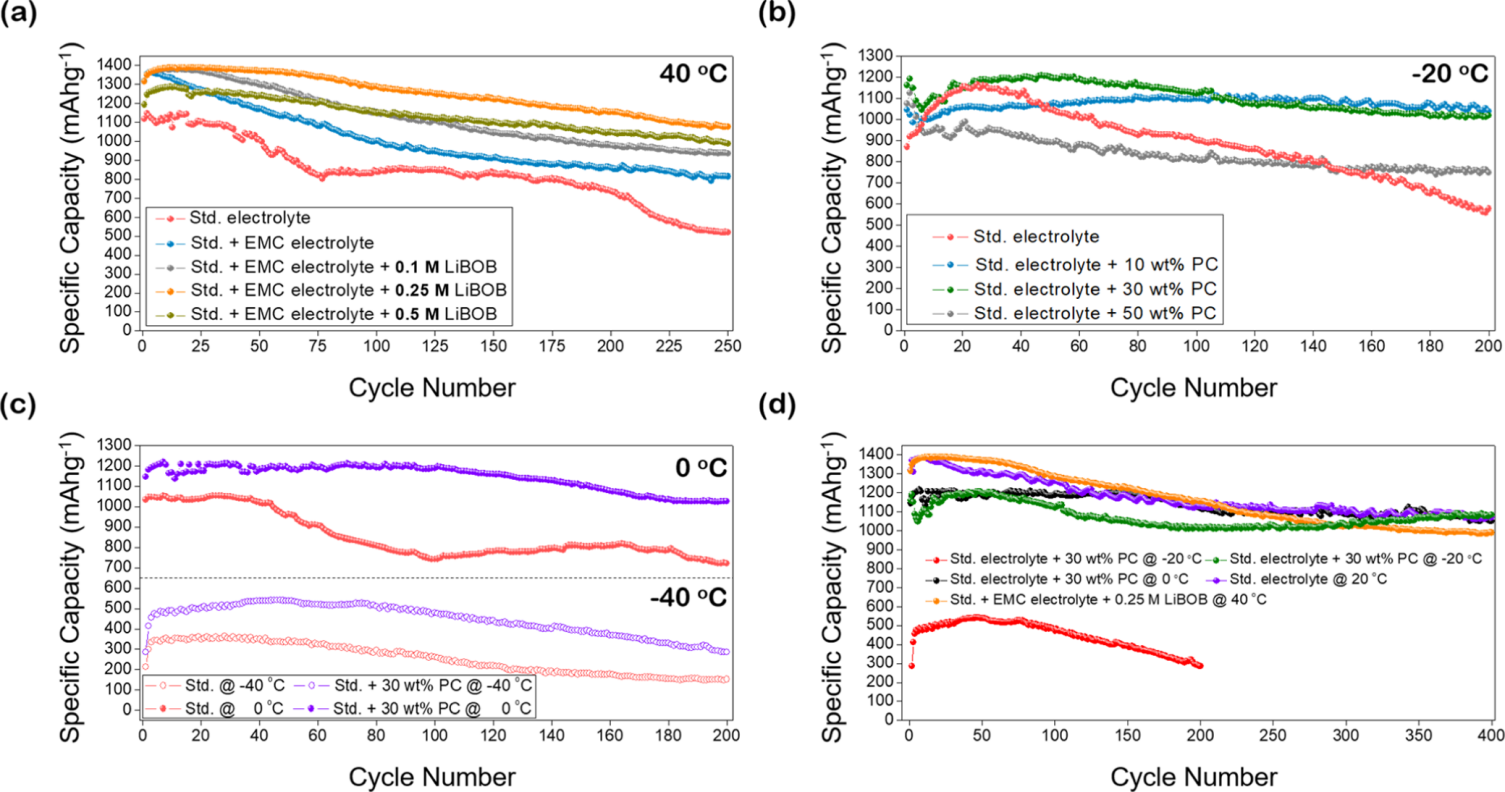
(a) LiCoO_2_||Ge cells cycled at 40 °C varying the EMC/LiBOB content in the electrolyte. (b) LiCoO_2_||Ge cells cycled at −20 °C varying the PC content of the electrolyte. (c) Discharge capacity of LiCoO_2_||Ge cells at −40 and 0 °C with the std. electrolyte (red) and std. electrolyte + 30 wt % PC (purple). (d) Long-term cycling of LiCoO_2_||Ge cells at −40 °C (red), −20 °C (green), 0 °C (black), 20 °C (purple), and 40 °C (orange) with optimized electrolytes. All cells were cycled galvanostatically at a 1C rate.

Modifications to the std. electrolyte with temperature-specific additives led to dramatic improvements in LiCoO_2_||Ge capacity retention at both high and low temperatures. First, EMC and LiBOB were chosen as high-temperature additives due to their performance-enhancing abilities in graphite-based cells at high temperatures.^9,16^Figure 3a illustrates the effect that varying the EMC and LiBOB content of the electrolyte has on cell stability. Initially, transitioning from a binary EC–DEC solvent to a ternary EC–DEC–EMC solvent stabilized capacity retention. In Figure 3a, the std. cell (red) retained 46.62% of its initial capacity after 250 cycles. Upon addition of EMC (blue), the capacity retention rose to 61.35%. Moreover, introducing EMC to the binary solvent mixture saw an increase in the average coulombic efficiency (CE) from 94.26 (red) to 99.68% (blue). The addition of LiBOB as an additive to the LiPF_6_-based electrolyte was found to notably improve capacity retention at 40 °C. LiBOB was tested as an additive at different molar concentrations (0.1, 0.25, and 0.5 M). Initially, a 0.1 M addition of LiBOB to the std. + EMC electrolyte (gray) improved capacity retention from 61.35 to 67.85%. A 0.25 M LiBOB addition (orange) proved to be the optimal composition, showing a capacity retention of 81.81% over the first 250 cycles. Increasing the LiBOB concentration further from 0.25 to 0.5 M (gold) saw a reduction in the maximum specific capacity from 1383 to 1289 mA h g^–1^ and a slight drop in capacity retention to 79.29%. The optimized 0.25 M LiBOB electrolyte (orange) improved the capacity fade rate threefold. The capacity of the std. cell (red) faded at a rate of 0.22% cycle^–1^, whereas the optimized “std. + EMC + 0.25 M LiBOB” cell (orange) exhibited a capacity fade rate of 0.07% cycle^–1^.

To realize better low-temperature performance, PC was introduced to the standard electrolyte configuration. Figure 3b illustrates the effect that varying the PC content has on the capacity and long-term stability of LiCoO_2_||Ge cells cycled at −20 °C. Solutions of std. electrolyte + 10 wt % (blue), 30 wt % (green), and 50 wt % (gray) PC were tested and compared to the standard cell (red). The best-performing electrolyte compositions were found to be 10 wt % and 30 wt % PC, achieving 93.42 and 89.94% capacity retention after 200 cycles, respectively. Increasing the PC content further to 50 wt % led to a reduction in the overall cell capacity and accelerated capacity fade, retaining 69.65% capacity after 200 cycles. The poor performance of the PC-free cell can be related to the inability of the binary electrolyte to suppress electrolyte freezing at −20 °C. Images taken of the standard electrolyte and the standard electrolyte + 30 wt % PC show that at −20 °C, PC actively mitigates electrolyte freezing, which in turn promotes better cell performance (Figure S7a,b).

To analyze the versatility of this low-temperature electrolyte, its performance-enhancing ability was tested over a wider low-temperature range. In Figure 3c, the standard electrolyte cell (red) was tested versus the optimized 30 wt % PC cell (purple) at −40 and 0 °C. Similar to cells cycled at −20 °C, the introduction of PC to the electrolyte solution improves cell performance at −40 and 0 °C. At −40 °C, 30 wt % PC raised the max. capacity of the cell from 359 mA h g^–1^ (red) to 551 mA h g^–1^ (purple) and improved capacity retention from 40.11 to 54.79% after 200 cycles. At −40 °C, PC does not completely suppress electrolyte freezing (Figure S7c), leading to an increase in internal resistance and hindered Li-ion transport through the electrolyte. However, even at −40 °C, the PC-containing cell reaches 551 mA h g^–1^, 1.5× higher than the theoretical capacity for graphite @ RT. At 0 °C, the 30 wt % PC-containing cell increased capacity retention from 69.09 to 86.91%, over 200 cycles, slowing the capacity fade rate from 0.15 to 0.06% cycle^–1^.

Extended cycling of LiCoO_2_||Ge cells from −40 to 40 °C with the temperature-specific optimized electrolytes is shown in Figure 3d. Collectively, the discharge plots present the best-performing LiCoO_2_||Ge cells, employing the optimized electrolyte compositions for that temperature. At −20 °C (green), LiCoO_2_||Ge can function impressively at a 1C rate, reaching 1207 mA h g^–1^ with a capacity retention of 90.42% over 400 cycles. LiCoO_2_||Ge cells showed a similarly high capacity retention at 0 °C (black), retaining 87.11% after 400 cycles. LiCoO_2_||Ge cells with optimized electrolytes cycled at 20 °C (purple) and 40 °C (orange) retained a capacity of 82.08 and 64.51%, respectively. An interesting trend was observed as the cell temperature was reduced from 40 to −20 °C; the capacity retention improved. After 400 cycles, LiCoO_2_||Ge cells at 40, 20, 0, and −20 °C reached respective capacity retentions of 64.51, 82.08, 87.11, and 90.42%. Consequently, the rate of capacity fade was lowered with the lowering of cell temperature. This improved capacity retention at lower temperatures can be attributed predominantly to temperature-dependent Ge lithiation and delayed amorphization at low temperatures, which is analyzed later. Corresponding charge capacities and CEs for LiCoO_2_||Ge cells from −40 to 40 °C are given in Figure S8. Furthermore, voltage profiles for the 1st, 2nd, and final cycle of each cell are outlined in Figure S9.

Parallel testing of LiCoO_2_||C cells with these temperature-specific electrolytes yielded no notable improvements to cell performance (Figure S10). The inability of PC to improve the low-temperature performance of graphite-based cells can be attributed to the destructive behavior of PC on graphite. PC promotes solvent co-intercalation into the graphite structure, leading to poor performance and graphite decomposition.^48,49^ Even with PC, charging and discharging of LiCoO_2_||C cells are practically infeasible below −20 °C, returning 10% of *Q*_th_ at −20 °C and 3% at −40 °C. The addition of EMC and LiBOB showed minimal improvement in LiCoO_2_||C capacity retention, increasing from 57.02 to 61.04%. The instability of the LiBOB-derived cell at 40 °C and the inability of PC to improve low-temperature performance suggest that the poor performance of graphite-based cells at temperature extremes cannot be easily rectified by modifying the electrolyte composition. Corresponding specific energies of LiCoO_2_||Ge and LiCoO_2_||C cells are depicted in Figure S11. At RT, LiCoO_2_||Ge delivers 174 W h kg^–1^ after 100 cycles, a 2× increase over that of LiCoO_2_||Ge (83 W h kg^–1^) at the same temperature. This disparity in RT specific energy of both cell types is further heightened at low temperatures, with LiCoO_2_||Ge returning a specific energy of 82 W h kg^–1^ at −40 °C, compared to 5 W h kg^–1^ for LiCoO_2_||C at −40 °C and equivalent to the specific energy of LiCoO_2_||C at RT (84 W h kg^–1^).

**Figure 4:**
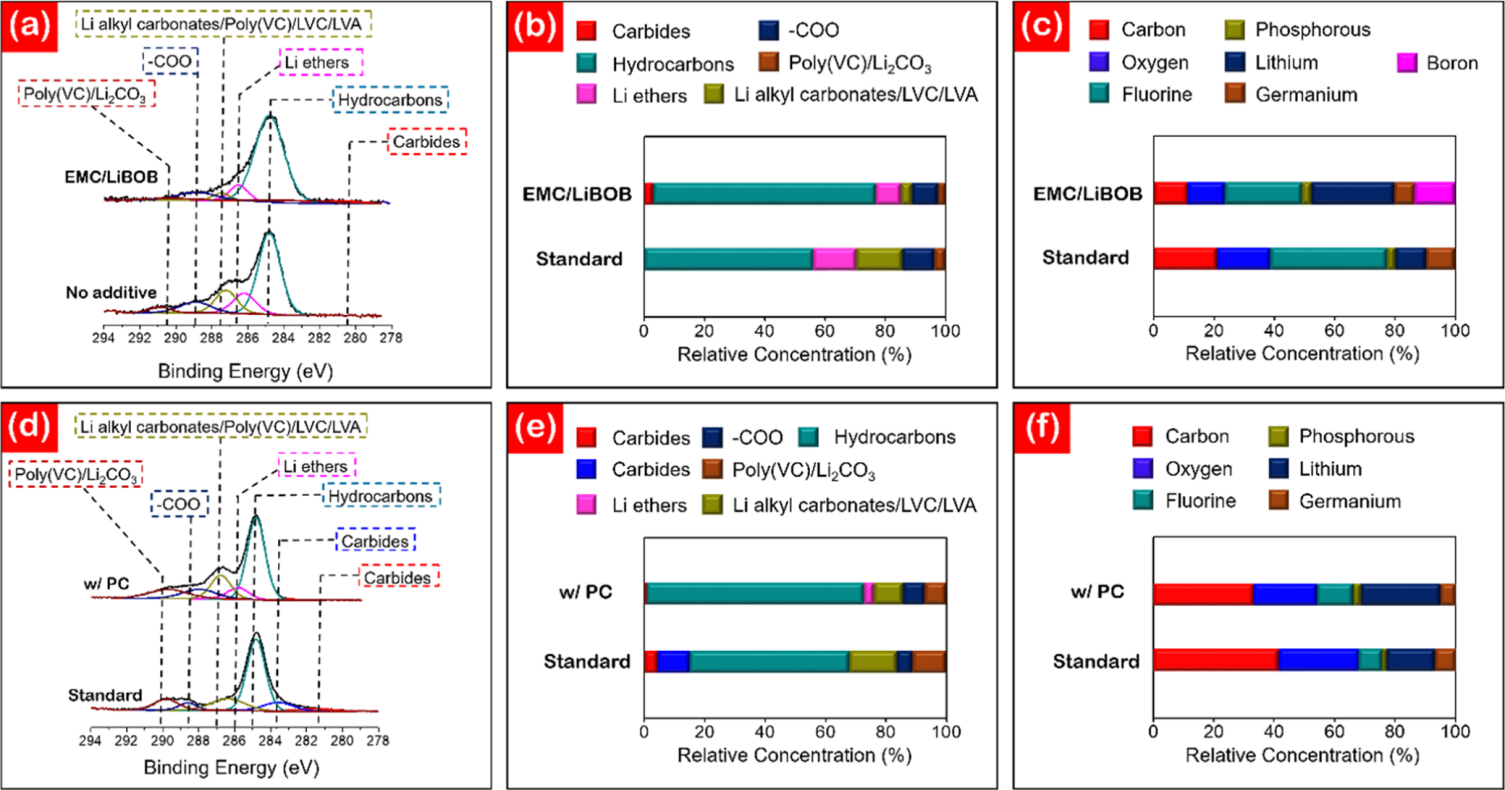
(a–c) XPS analysis of the effect of EMC/LiBOB on the SEI layer composition at 40 °C. (a) High-resolution C1s spectra, (b) relative concentrations of chemical species from C1s spectra, and (c) elemental concentrations from the survey scan. (d–f) XPS analysis of the effect of PC on the SEI layer composition at −20 °C. (d) High-resolution C1s spectra, (e) relative concentrations of chemical species from C1s spectra, and (f) elemental concentrations from the survey scan.

To examine the stabilizing effects of the high- and low-temperature additives, X-ray photoelectron spectroscopy (XPS) was employed to analyze the composition of the SEI. Ex situ XPS was employed to realize the exact chemical composition of the SEI layer and determine how the composition varied with the introduction of EMC/LiBOB and PC (Figure 4). XPS analysis of the effect of cycling temperature on the SEI composition using a standard electrolyte is given in Figure S12. First, XPS was carried out on Ge anodes after 25 cycles at 40 °C with and without EMC/LiBOB (Figure 4a–c). The relative concentrations of the chemical species in C1s spectra in (a) are quantified in (b). From the deconvoluted C1s spectra in (a), both SEI layers are composed predominantly of hydrocarbons (284.8 eV),^50^ Li ethers (286.2 eV),^51^ Li alkyl carbonates (287.2 eV),^52^ carboxylates/oxalates (288.9 eV),^53^ and Li_2_CO_3_/Poly(VC) (290.1 eV).^54^ In Figure 4a, the C1s spectra show concurrent signals for the Li_2_CO_3_/Li alkyl carbonates and poly(VC) peaks due to the carbon atoms existing in similar three-oxygen environments.^52^ Analysis of the deconvoluted O1s spectra for the two anode types shows the strong presence of a peak at 534 eV, indicative of poly(VC) incorporation into the SEI layer^55^ (Figure S13a). Poly(VC) is widely known as a vital component in the SEI layer, enhancing stability and flexibility of the passivation film.^53,56−58^ From the C1s spectra, the standard and the EMC/LiBOB-containing anodes do not differ drastically in their chemical makeup. The most notable difference is the emergence of a carbide B–C peak at 281 eV in the EMC/LiBOB-derived film, indicative of boron incorporation into the SEI layer.^59^ The analogous B–C peak can be observed at 189.1 eV in the deconvoluted B1s spectra shown in Figure S13c. The incorporation of boron into the SEI layer is demonstrated by the elemental composition data in Figure 4c. The incorporation of LiBOB as an additive to the LiPF_6_-based electrolyte reduced the overall fluorine content from 38.7 to 25.6% with boron accounting for 13.4% of the SEI layer upon LiBOB addition. Moreover, the F1s spectra show a higher LiF content for the EMC/LiBOB-containing cell, indicative of a more stable SEI layer^60,61^ (Figure S13b). A combination of partial fluorine substitution, boron incorporation, and greater LiF production collectively contributes to the enhanced capacity retention by stabilizing the SEI layer.

Figure 4d–f quantifies the effect of introducing PC into the electrolyte mixture for cells cycled at −20 °C. The most notable difference between the two anode types in the C1s spectra is the presence of carbide species in the PC-free SEI layer (Figure 4d,e). Two peaks are present in the PC-free anode, a minor peak at 281.5 eV and a large peak at 283.8 eV, corresponding to carbide species.^62^ Carbide formation at low temperatures can be attributed to lithium decomposition at the anode’s edge.^63^ The introduction of PC was found to largely suppress carbide formation and reduce the relative concentration of the carbonate-containing species, indicative of less lithium being consumed at the anode surface. Additionally, PC was found to promote Li ether incorporation in the SEI layer. At −20 °C, Li ether is not found in the standard SEI (Figure 4d,e). In Figure 4f, the addition of PC did not lead to major variations in the elemental composition, as its addition does not introduce any additional elemental species. A notable difference between the standard and the PC-containing SEI layers is the concentration of the respective poly(VC) signals. The O1s and F1s spectra for the PC-free and PC-containing cells are shown in Figure S14. In the O1s spectra, poly(VC) accounts for 15.8% of the oxygen content in the standard SEI, which rises to 41.8% in the PC-containing SEI (Figure S14a,c). Additionally, from the F1s spectra (Figure S14b,d), the PC-containing SEI layer shows a higher LiF concentration than the standard SEI (75.4% compared to 51.9%). Enhanced low-temperature performance of PC-containing cells (as shown in Figure 3b) can therefore be collectively attributed to improving the stability of the SEI layer and suppressing electrolyte crystallization.

**Figure 5:**
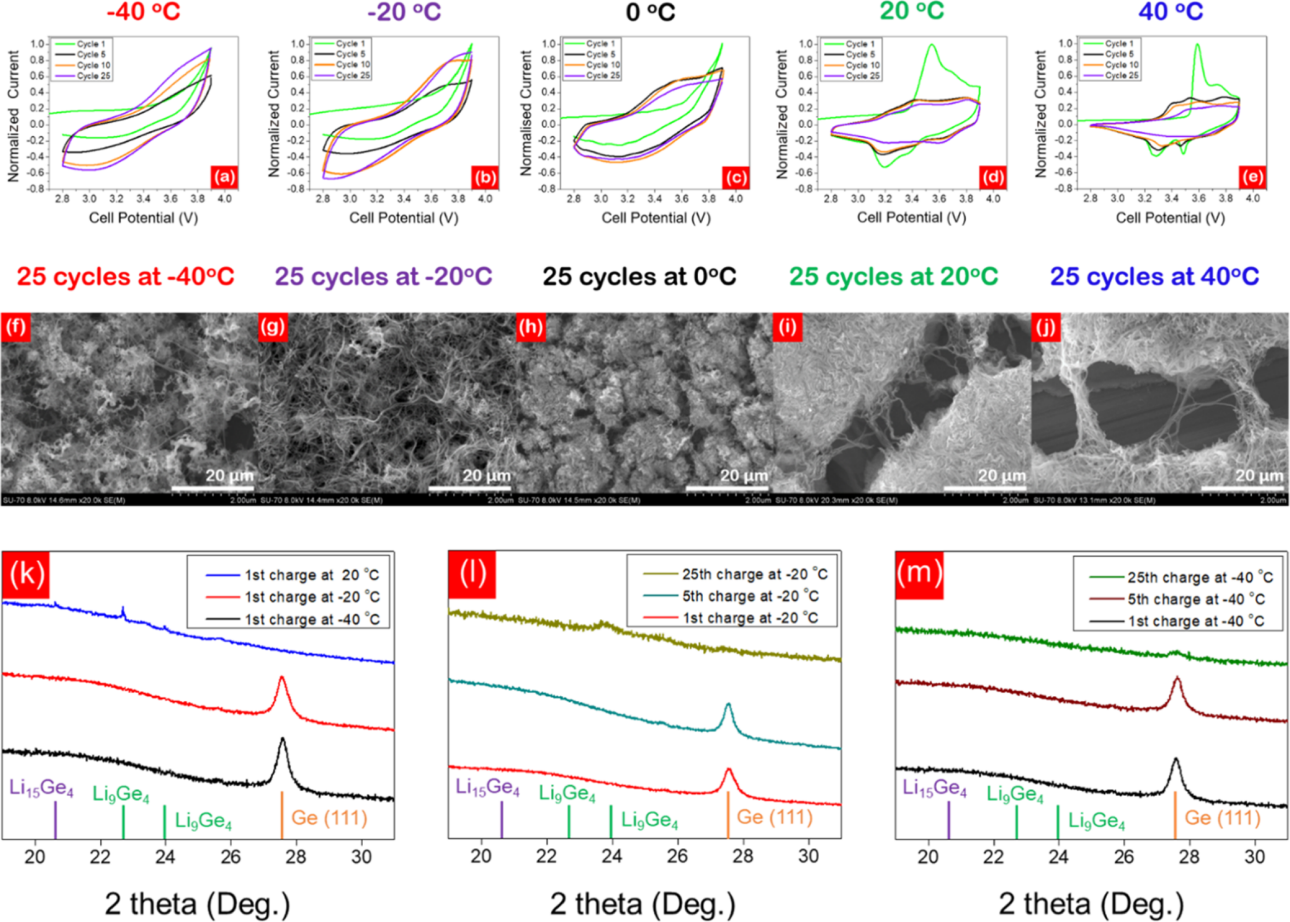
CVs of LiCoO_2_||Ge cells at (a) −40, (b) −20, (c) 0, (d) 20, and (e) 40 °C. Cells were cycled at a scan rate of 0.05 mV s^–1^ between 2.8 and 3.9 V. SEM images of Ge NWs after 25 cycles at (f) −40, (g) −20, (h) 0, (i) 20, and (j) 40 °C. Stacked X-ray diffraction patterns of Ge NWs (k) after the 1st charge at −40 °C (black), −20 °C (red), and 20 °C (blue), (l) after the 1st (red), 5th (light blue), and 25th (gold) charge at −20 °C, and (m) after the 1st (black), 5th (brown), and 25th (green) charge at −40 °C. The reference patterns for Li_9_Ge_4_ (green; JCPDS card no. 73-2487), Li_15_Ge_4_ (purple; JCPDS card no. 89-3034), and Ge (orange; JCPDS card no. 89-5011) are represented by the bars at the bottom of the graphs. The std. electrolyte was used for each test.

A combination of SEM, cyclic voltammetry (CV), and X-ray diffraction (XRD) was employed to elucidate the underlying mechanistic behavior of temperature-controlled morphology evolution of crystalline Ge NWs to a porous Ge framework (Figure 5). CV and SEM were utilized to track morphological changes in the Ge active material during cycling to determine whether the evolution of the porous network morphology is temperature-dependent. CV scans were taken from −40 to 40 °C, in 20 °C increments (Figure 5a–e). Cells were cycled for 25 cycles at a scan rate of 0.05 mV s^–1^. SEM images were taken of the corresponding Ge anodes after 25 cycles (Figure 5f–j). For consistency, the standard electrolyte was used throughout testing. Cells cycled at (a) −40, (b) −20, and (c) 0 °C all exhibit a gradual widening of the CV curves and an increase in peak intensity as the cell cycles. This curve widening is most prominent at −40 and −20 °C but still apparent at 0 °C. This gradual increase in peak intensity demonstrates the hindered lithiation/delithiation mechanics and slow activation of cell capacity at low temperatures. Conversely, cells cycled at (d) 20 and (e) 40 °C exhibit the characteristic electrochemical behavior for Ge-based cells. The 1st cycle involves a high degree of Li consumption and irreversible capacity loss to form the SEI layer. Successive cycles suffer reductions in peak intensity as cell capacity gradually decays. From the SEM images, a clear distinction is noted in the evolution of the Ge morphology. To better understand the transformation of individual NWs to discrete interconnected Ge islands, SEM images of pristine Ge NWs are provided in Figure S15. The characteristic porous Ge network^34^ is achieved after 25 cycles at (i) 20 and (j) 40 °C. As the cell temperature is lowered, the morphology of the anode approaches that of uncycled Ge NWs. At −40 °C, individual Ge NWs can still be seen after 25 cycles. SEM images are included to highlight morphological differences in post-cycled Ge NWs at temperature extremes, rather than incremental differences from temperature to temperature. Although variations in the post-cycled morphology of Ge NWs are not quantitative of phase change, they do highlight the impact of low temperatures in delaying mesh formation. The slow capacity activation of cells cycled at low temperatures combined with delayed morphology evolution highlights the critical role cell temperature plays in the performance of alloy-type anodes. Parallel testing of LiCoO_2_||Ge cells with optimized electrolytes revealed a similar trend in Ge morphology (Figure S16), suggesting that the evolution of the Ge morphology is driven predominantly by cell temperature rather than electrolyte composition.

XRD analysis of lithiated Ge anodes [charged to 3.9 V at 0.05 C for 1 cycle at −40 °C (black), −20 °C (red), and 20 °C (blue)] revealed the temperature-dependent crystalline–amorphous phase transition of Li alloying materials (Figure 5k–m). At 20 °C, the crystalline–amorphous phase transition occurs during the first cycle, as evidenced by the attenuation of the Ge (111) peak and the emergence of Li_*x*_Ge peaks. Peaks at 22.7 and 23.9° represent the Li_9_Ge_4_ phase, whereas the peak at 20.6° corresponds to the higher order Li_15_Ge_4_ phase. On the other hand, at low temperatures, Ge amorphization is greatly suppressed. At −40 and −20 °C, crystalline Ge persists after the 1st charge cycle. Moreover, XRD reveals no apparent Li_*x*_Ge phases. After five cycles at −20 °C, Ge (111) can be still observed (Figure 5l). The crystalline Ge peak disappears after 25 cycles, accompanied by the emergence of a low-order Li_9_Ge_4_ peak at 23.9°. Analogous testing was carried out for Ge cycled at −40 °C (Figure 5m). Similar to −20 °C, the crystalline Ge (111) peak is present after five cycles. However, extended cycling found that the Ge (111) peak persists after 25 cycles, with no apparent Li_*x*_Ge peaks visible. This agrees well with Figure 5f, which shows the presence of individual intact Ge NWs after 25 cycles at −40 °C. Low temperatures delay Ge amorphization by suppressing lithiation and this behavior is intensified as the temperature is further lowered. The results explain the trends in Figure 5 where a slow capacity activation and delayed morphology evolution at low temperatures are attributed to suppression of Ge lithiation and amorphization. This delayed lithiation behavior of alloy-type materials bodes well for long-term stable cycling at low temperatures. From Figure 5l, cells cycled at −20 °C for 25 cycles exhibit no high-order Li_15_Ge_4_ phase with only the Li_9_Ge_4_ phase present after 25 cycles. Low temperatures may behave as a natural capacity limitation, setting an upper limit on cell capacity, suppressing complete lithiation of the Ge active material, and enhancing the long-term low-temperature stability of LiCoO_2_||Ge cells.

**Figure 6:**
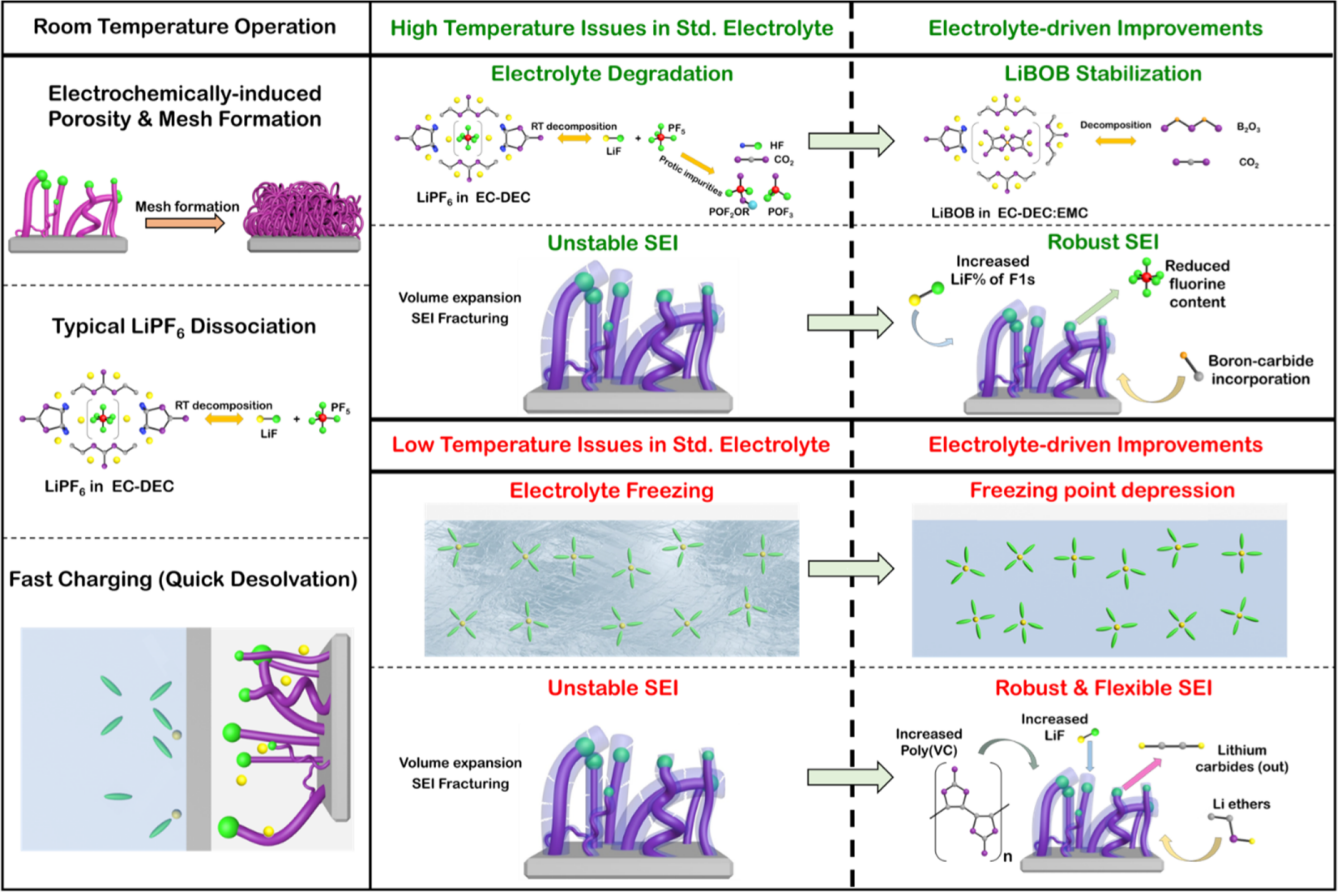
Schematic summary of the temperature-dependent behavior of Ge NW-based cells. The high-temperature electrolyte instability and the low temperature freeing are overcome through modifications to the std. electrolyte, dramatically enhancing both cell capacity and capacity retention.

The electrolyte-driven enhancements of LiCoO_2_||Ge performance at both high and low temperatures are summarized in Figure 6. Rapid amorphization and mesh formation at RT improve SEI stability by generating a porous framework to facilitate large volume expansion during charge. At RT, stable dissociation of LiPF_6_ into LiF and PF_6_ aids SEI formation. A combination of short diffusion lengths and fast Li-ion desolvation mechanics promotes fast charging/discharging of Ge NWs at RT. HF/POF_3_ formation and gas evolution at high temperatures destabilize the cell through unstable SEI formation and eventual current collector corrosion. The dual addition of EMC and LiBOB improved high-temperature capacity retention dramatically by suppressing harmful byproduct formation through boron substitution and improved the stability of the SEI by increasing the LiF percentage of the fluorine composition and lowering the overall fluorine content. At low temperatures, the std. electrolyte freezes below −20 °C, dramatically affecting lithiation/delithiation. The introduction of PC played a dual role in suppressing electrolyte freezing and enhancing the flexibility and strength of the as-formed SEI through increases in the LiF and poly(VC) content and the incorporation of Li ethers and the suppression of lithium carbides. Modifying the electrolyte composition for temperature-specific operation can dramatically enhance the performance of Ge NW-based cells. Ensuring the stability of the electrolyte and the SEI layer facilitates stable cycling over the entire range of EV operation and beyond.

## Conclusions

In this study, Ge NW-based Li-ion full cells were demonstrated to be extremely robust over a temperature range of −40 to 40 °C. The LiCoO_2_||Ge NW cells in a “standard” electrolyte adapted well to variations in cell temperature, showing high capacity and impressive cycling stability and vastly outperformed LiCoO_2_||graphite cells under the same conditions. Temperature-specific additives were shown to play a further role in enhancing the high- and low-temperature stability of LiCoO_2_||Ge cells. Propylene carbonate dramatically improved capacity retention at low temperature, by concurrently enhancing SEI flexibility and suppressing electrolyte crystallization. A binary additive mixture of EMC and lithium bis(oxalato)borate stabilized LiCoO_2_||Ge cells at elevated temperatures, through partial fluorine/boron substitution. This stabilizing behavior experienced by LiCoO_2_||Ge cells was not shown by LiCoO_2_||C cells, with electrolyte modifications showing no improvement in cell performance. The temperature-dependent performance of LiCoO_2_||Ge cells was further explained by the temperature-specific evolution of the anode morphology during cycling. We showed that delayed amorphization dramatically improved capacity retention by limiting complete lithiation during charge. The results show that alloying anodes in the form of LiCoO_2_||Ge cells function admirably at the extremes of temperatures experienced in transport (−40 to 40 °C), exhibiting excellent stability and high capacity and dramatically outperforming LiCoO_2_||C cells under the same conditions.

## Experimental Section

### Sample Preparation

Graphite slurries were prepared via a typical slurry processing technique. Graphite anodes consisted of 90 wt % graphite, 5 wt % carbon black, and 5 wt % carboxymethyl cellulose (CMC). Carbon black was homogeneously dispersed in a 1.5 wt % solution of CMC in H_2_O. The mixture was left to stir for 6 h. Graphite was added slowly and incrementally, with constant stirring to ensure homogeneity of the slurry mixture. The slurry was left to stir overnight prior to casting. Cu foil (purchased from Pi-Kem), with a thickness 9 μm, was used as the current collector. The slurry mixture was cast on the Cu foil using a heated vacuum doctor blade. A blade height of 25 μm achieved the desired 1.74 ± 0.13 mg cm^–2^ anode mass loading. The anode tape was dried under vacuum for 12 h@120 °C. Prior to cell assembly, anodes with an area of 0.64 cm^2^ were punched out and stored in an Ar-filled glovebox.

A previously published, rapid solvent-free protocol was employed for high-density growth of germanium NWs via a vapor–solid–solid (VSS) mechanism (Figure S17).^64^ Briefly, the reaction proceeds through thermal decomposition of the organometallic germanium precursor, diphenylgermane (DPG). Ge NWs were grown on stainless steel (SS), allowing them to be used directly as battery anodes. SS foil (purchased from Pi-Kem Ltd.) with a thickness of 0.1 mm was sanded prior to evaporation to increase the surface area and improve adhesion of the thermally evaporated catalytic film. The P600 grit sandpaper was used in the sanding process. Afterward, the SS substrates were washed in acetone to remove residual particles. Substrates with an area of 0.64 cm^2^ were punched out and a 2 nm coating of Cu (Kurt J. Lesker 99.999%) was thermally evaporated onto the SS substrate. The substrate was placed on a Stuart CD162 hotplate/digital stirrer at 420 °C in an argon-filled glovebox. To contain the decomposing vapor, a SS confiner was placed over the substrate. The Ge precursor was dropcast onto the substrate, by injecting it through the top of the confiner. High-density growth of Ge NWs was achieved after 30 s. The mass loading of the Ge NW substrates was found to correlate directly to the volume of the precursor used. An appropriate amount of DPG (2–10 μL) was dropcast onto the SS substrate, depending on the desired anode mass loading (0.25–0.62 mg cm^–2^). Anodes were stored under argon prior to cell assembly to minimize native oxide growth.

### Cell Assembly and Electrochemical Analysis

Two-electrode Swagelok-type cells were assembled in an Ar-filled glovebox. LCO cathode tapes were purchased from NEI Corporation with an active mass loading of 6.2 mg cm^–2^. Cathodes with an area of 0.64 cm^2^ were punched out. No further treatment of the Ge or graphite anodes was required prior to cell assembly. Both anode types were matched in terms of areal capacity with a constant PN ratio for both cell types (LiCoO_2_||C and LiCoO_2_||Ge). A PN ratio of 1.5 was kept constant throughout testing. The PN ratio denotes the capacity ratio between the positive (P) and negative (N) electrodes. Previous work by our group highlighted the importance of capacity balancing for full-cell configurations.^65^ For PN1.5 cells, the active mass loadings of Ge and graphite were set at 0.42 and 1.57 mg cm^–2^, respectively. The electrodes were separated from each other using a porous polypropylene Celgard separator paper. A total of 1 M LiPF_6_ in EC–DEC (1:1 v/v) + VC (3 wt %) was chosen as the standard (std.) electrolyte throughout testing with modifications made to the solution for high- and low-temperature cycling. The electrochemical performances of these cells were analyzed through galvanostatic cycling within a 2.8–3.9 V potential window, using a BioLogic MPG-2 multichannel potentiostat. For low-temperature cycling (−40 to 0 °C), the PC content of the electrolyte was varied. Electrolyte solutions (1 M LiPF_6_ in EC–DEC (1:1) + 3 wt % VC) with 0, 10, 30, and 50 wt % PC were made up and tested as potential electrolyte compositions for low-temperature cycling. EMC and LiBOB were tested as high-temperature additives. Initially, 1 M LiPF_6_ in EC–DEC–EMC (1:1:1) + 3 wt % VC was formulated and denoted as “std. + EMC”. LiBOB was tested in different molar concentrations (0.1, 0.25, and 0.5 M) by dissolving it in a ternary EC–DEC–EMC (1:1:1) mixture and incorporating it into the std. + EMC solution.

Analogous to rate capability testing, temperature capability testing increased cell temperature stepwise, analyzing the electrochemical response of the cell to variations in cell temperature. Using a combination of freezers and incubators, cells were first cycled for five cycles at −40 °C. After five cycles, temperature was raised to −20 °C and the cell was left idle on discharge overnight to allow for temperature equalization. This was repeated up as far as 40 °C, with the temperature increased in 20 °C increments after every five cycles. Cell response was analyzed by plotting discharge capacity as a function of cell temperature from −40 to 40 °C. EIS was carried out using Biologic BT-Lab V1.65 software. Cells were subjected to low current slow partial charge to 3.7 V to stabilize the open-circuit potential. The cells were subsequently allowed to relax to the open circuit, until the potential drift became low enough to remove DC bias. The EIS spectra were then recorded with a peak amplitude of 10 mV, between 20 mHz and 100 kHz. Equivalent circuit models were then fitted to the EIS data using the EIS fitting function of Biologic BT-Lab V1.65 software.

### Ex Situ Characterization

SEM was carried out using a Hitachi SU-70 system with an accelerating voltage of 8 kV. The cells were disassembled in an Ar-filed glovebox. The electrode was soaked in acetonitrile overnight before being successively washed in 0.1 mM acetic acid, deionized water, and ethanol. This process removes the SEI layer on the anode surface along with any lingering electrolyte material and residual lithium content.^66^ XRD was achieved using a PANalytical X’Pert PRO MRD with a radiation source consisting of Cu Kα (λ = 1.5418 Å) and an X’celerator detector. To analyze lithiated Ge anodes under XRD, atmospheric oxidation had to be kept at a minimum. This was achieved by taping the Ge anodes to glass slides using Scotch tape. The tape ensured that the Ge anodes remained in the charged state and removed contact with the atmosphere. Background scans of the blank glass slide with Scotch tape allowed for the identification of crystalline peaks associated with the samples. XPS was carried out using the Kratos AXIS ULTRA spectrometer with Al Kα 1486.58 eV. Prior to XPS, substrate pre-treatment involved washing off the residual electrolyte by immersing the anode in dimethyl carbonate (DMC) for 1 h and allowing it to dry in an Ar environment. The core level binding energies were determined by taking the charge reference of C1s at 284.8 eV. The narrow regions were analyzed using 20 eV with the survey spectra analyzed with a pass energy of 160 eV. The broad peaks were fitted using a Gaussian–Lorenzian mixed-type fit with the narrow regions constructed using a Shirley-type background.

## Supporting Information Available

Performance comparison of LiCoO_2_||Ge NWs to published work of similar materials, first-cycle DCPs of LiCoO_2_||Ge cells, temperature capability testing of LiCoO_2_||Ge (red) and LiCoO_2_||C (blue) cells, and Nyquist plots of LiCoO_2_||C cells and LiCoO_2_||Ge cells (PDF)

## Abbreviations

CE: coulombic efficiency
CMC: carboxymethyl cellulose
CV: cyclic voltammetry
DCP: differential capacity plot
DEC: diethyl carbonate
DMC: dimeythl carbonate
DPG: diphenylgermane
EC: ethyl carbonate
EIS: electrochemical impedance spectroscopy
EMC: ethyl methyl carbonate
EV: electric vehicle
ICE: internal combustion engine
LiBOB: lithium bis(oxalato)borate
NW: nanowire
PC: propylene carbonate
*Q*_th_: theoretical capacity
RT: room temperature
RCT: rate capability testing
*R*_CT_: charge transfer resistance
SEI: solid electrolyte interphase
SS: stainless steel
TEM: transmission electron microscopy
VSS: vapor–solid–solid
XPS: X-ray photoelectron spectroscopy
XRD: X-ray diffraction
