# Supplementary material for: Alloying Germanium Nanowire Anodes Dramatically Outperform Graphite Anodes in Full-Cell Chemistries over a Wide Temperature Range
Source: ACS Appl Energy Mater. 2021 Feb 2;4(2):1793–804. doi: 10.1021/acsaem.0c02928 (PMC8288911; doi:10.1021/acsaem.0c02928)
Supplement: Supplementary file 1 — ae0c02928_si_001.pdf [file ae0c02928_si_001.pdf]

Alloying Germanium Nanowire Anodes Dramatically Outperform Graphite Anodes in Full Cell Chemistries over a Wide Temperature Range

Gearoid A. Collins[1], Karrina McNamara[2], Seamus Kilian[1], Hugh Geaney[1,] and Kevin M. Ryan[1,*]*

[1]Department of Chemical Sciences, University of Limerick, V94 T9PX, Ireland

[2]Department of Physics, University of Limerick, V94 T9PX, Ireland

Corresponding E-mail(s): hugh.geaney@ul.ie, kevin.m.ryan@ul.ie

Supporting Information

Table S1: Performance comparison of $LiCoO_2$||Ge NWs (this work) to published work of similar materials.

Material Type	Half-Cell	Full-Cell	LT Capacity	LT Stability	Ref.
Ge NWs		✓	1207 mAh g^{-1} @ -20 °C for 1C (87.2 % of RT capacity)	91 % after 400 cycles at -20 °C	Our work
Mesoporous Ge NPs		✓	90 mAh g^{-1} @ -20 °C for C/2 (87.4 % of RT capacity)	80 % after 50 cycles at -20 °C	[1]
Amorphous Si microparticles	✓		600 mAh g^{-1} @ -30 °C for C/4	N/A	[2]
Cu-Ge-Al nanoporous framework	✓		361 mAh g^{-1} @ -20 °C (0.1 mA g^{-1})	N/A	[3]
Microporous Cu-Zn alloy network	✓		197 mAh g^{-1} @ -20 °C (0.1 A g^{-1})	N/A	[4]
Sn NPs embedded in expanded graphite	✓		200 mAh g^{-1} @ -20 °C (30.8 % of RT capacity) for C/10	N/A	[5]

Sn NPs embedded in amorphous carbon		✓	105 mAh g^{-1} @ -5 ^{o}C for C/4 (91 % of RT capacity)	93 % after 210 cycles at -5 ^{o}C (C/4)	[6]

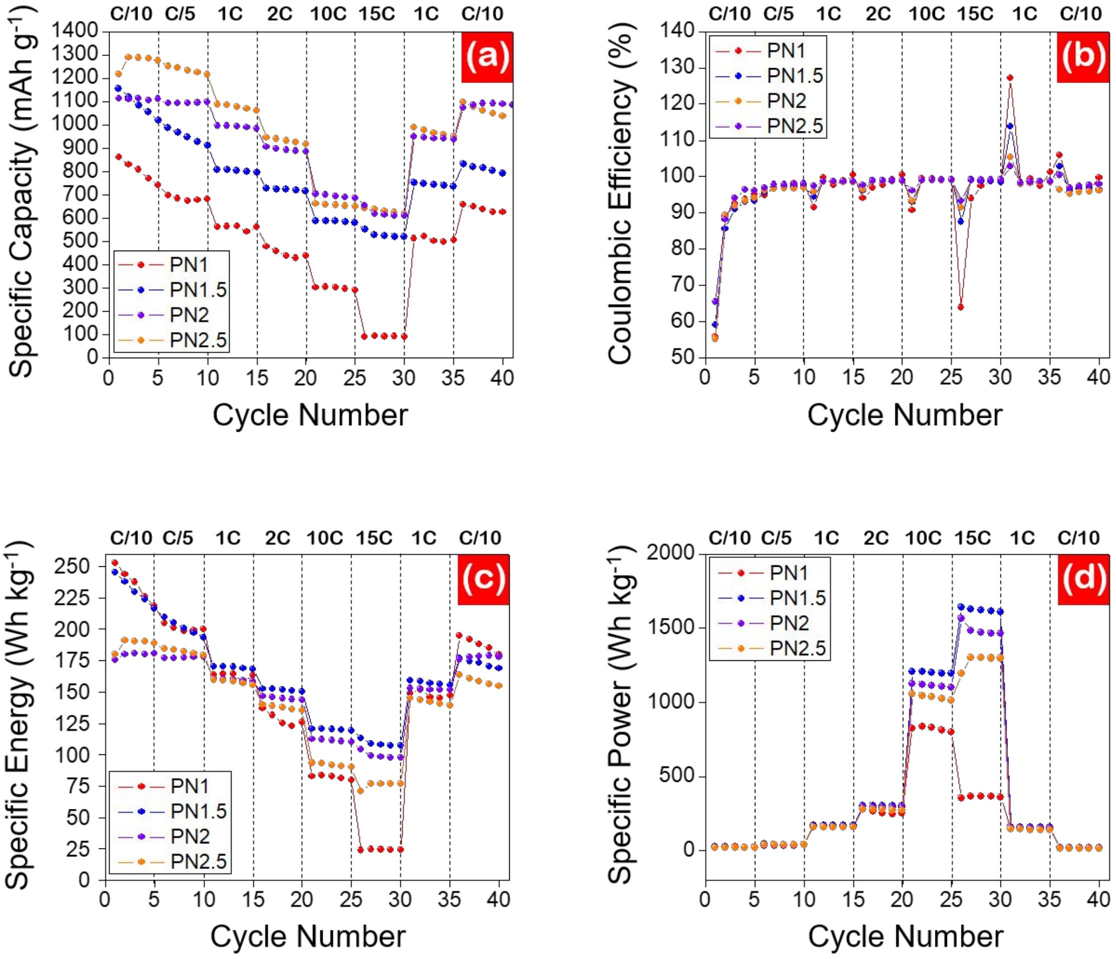

Figure S1. (a) RCT of $LiCoO_2$||Ge NW FCs for different PN ratios and corresponding (b) CE, (c) specific energy and (d) specific power accounting for active and inactive masses of both electrodes and. The PN ratio was adjusted by keeping the cathode active loading constant (6.2 mg cm^{-2}) and varying the anode loading from 0.25 mg cm^{-2} (PN2.5) to 0.63 mg cm^{-2} (PN1).

$LiCoO_2$||Ge cells with PN ratios ranging from PN1-PN2.5 were subjected to rate capability testing (RCT), quantifying the specific capacity, energy and power as a function of C-rate and PN ratio (Figure S1). Commercial LIBs typically employ a low PN ratio of 0.9-1.1, to maximise energy density by minimising inactive cathode weight. Such low PN ratios were found not to be commercially acceptable for Li alloying anode types. Intuitively, PN ratios close to unity offer the highest viable energy and power densities for Li alloying anode-based LIBs. However, analysing cell performance as a function of PN ratio reveals the superiority of PN1.5 over all other electrode matchings, maximising specific energy and power. Insufficient lithium of PN1 to regulate SEI growth and maintain high specific capacity, counteracts low cell weight, delivering the lowest specific energy and specific power of all cells tested. Comparatively, PN1.5 delivered the best overall performance, reaching max. specific energy and power values of 109 Wh kg^{-1} (Figure S1c) and 1650 W kg^{-1} (Figure S1d) at 15C, compared to 25 Wh kg^{-1} (Figure S1c) and 375 W kg^{-1} (Figure S1d) for PN1. The improved specific capacity of PN2 and PN2.5 cells over PN1 and PN1.5 (Figure S1a) comes at the cost of a reduced energy density (Figure S1c), attributed to excess cathode mass. Overall, PN1.5 was found to strike a balance between low weight (to maximise energy and

power densities) and excess Li (to extend cycle life after preliminary SEI-forming losses).

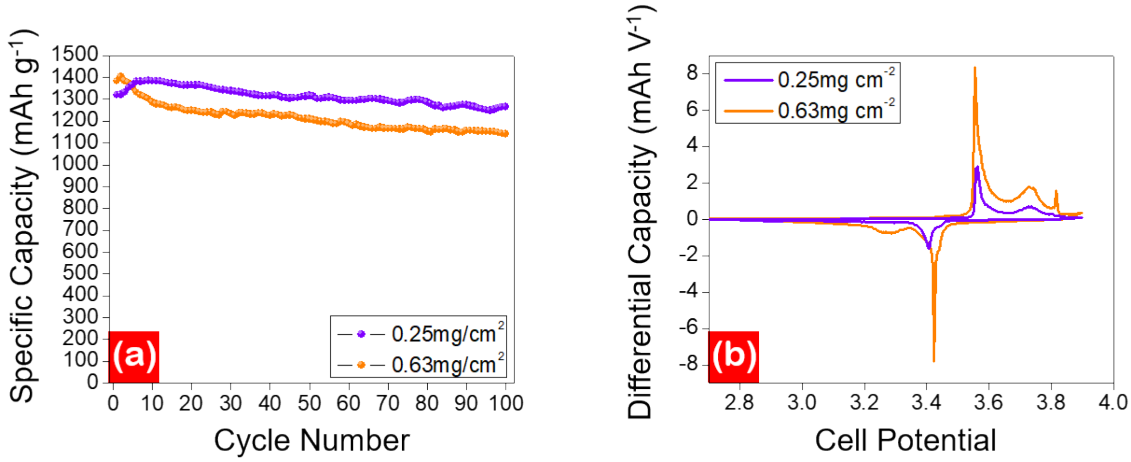

Figure S2. (a) Discharge capacity and (b) differential capacity plot (DCP) of $LiCoO_2$||Ge cells with a constant PN ratio of 1.5 with anode loadings of 0.25 mg cm^{-2} and 0.63 mg cm^{-2} (corresponding to LCO loadings of 3.71 mg cm^{-2} and 9.35 mg cm^{-2}). Such anode loadings mimic the anode loadings of PN2.5 and PN1 cells within the main testing set, respectively. Setting a constant PN ratio of PN1.5 isolates loading-driven capacity behaviour over the anode loading range (0.25-0.63 mg cm^{-2}).

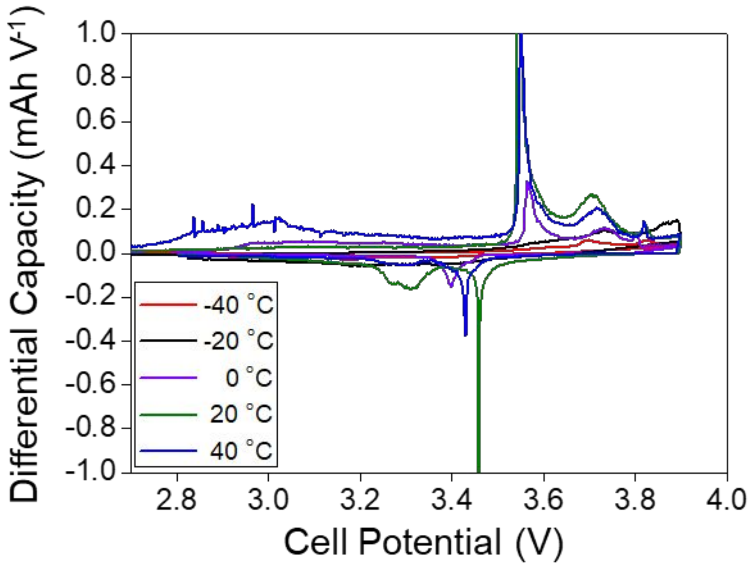

Figure S3. First cycle DCPs of $LiCoO_2$||Ge cells at (a) -40 °C, (b) -20 °C, (c) 0 °C, (d) 20 °C and (e) 40 °C in std. electrolyte. Cells were cycled at C/2 between 2.8-3.9 V.

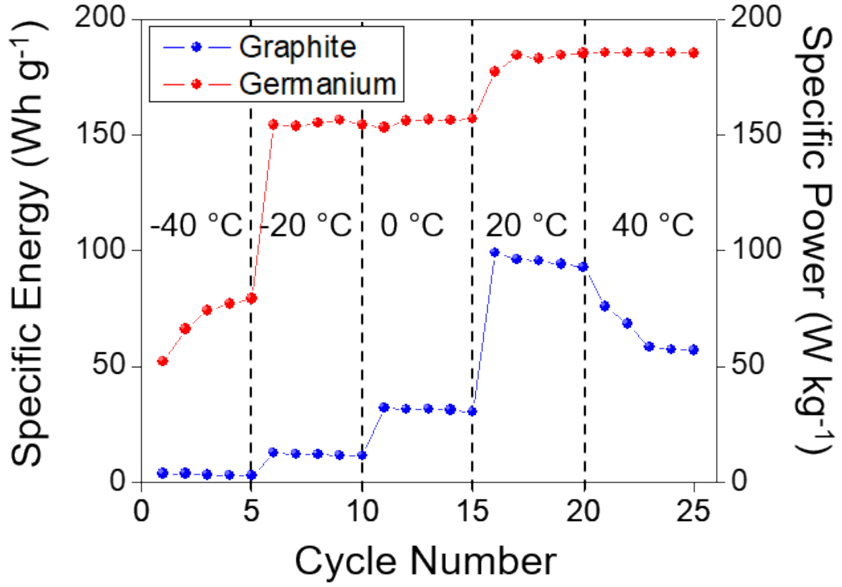

Figure S4. Temperature capability testing of $LiCoO_2$||Ge (red) and $LiCoO_2$||C (blue) cells with representative specific energy and specific power for PN1.5. Note, energy and power values are equivalent at a 1C rate.

Temperature capability testing of $LiCoO_2$||Ge and $LiCoO_2$||C cells with representative energy and power densities at a 1C rate is outlined in Figure S4. For anodes of equivalent areal capacities, $LiCoO_2$||Ge far outperforms $LiCoO_2$||C at every temperature. At RT, $LiCoO_2$||C delivers a reasonable 100 Wh kg^{-1}, compared to 185 Wh kg^{-1} for $LiCoO_2$||Ge. However, low temperatures severely degrade $LiCoO_2$||C performance, with $LiCoO_2$||Ge reaching 156 Wh kg^{-1} at -20 ^{o}C, a 13x increase over $LiCoO_2$||C at -20 ^{o}C (12 Wh kg^{-1}).

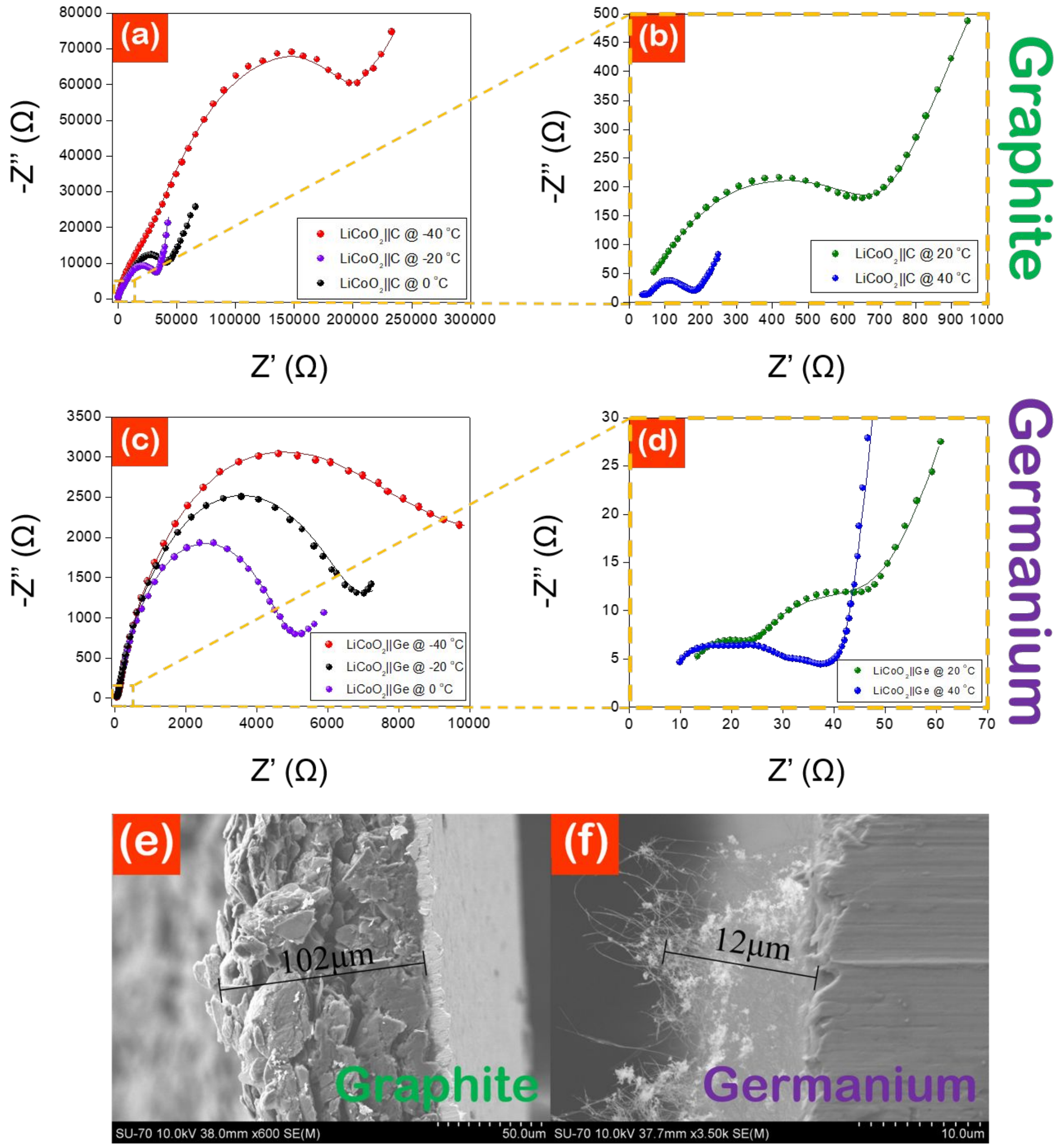

Figure S5. Nyquist plots of (a-b) $LiCoO_2$||C cells and (c-d) $LiCoO_2$||Ge cells between -40 °C and 40 °C. (b) and (d) represent the inset figures of (a) and (c), at the low impedance range. The solid circles depict the experimental data while the lines represent the fitting of the equivalent circuit to the data. Cross-sectional SEM of capacity-matched (e) graphite on Cu foil and (f) Ge NWs on SS.

Nyquist plots for $LiCoO_2$||C and $LiCoO_2$||Ge cells cycled between -40 ^oC and 40 ^oC are shown in Figure S5a-d. The plots were recorded during the fifth charge cycle of each cell. The data reflects the dramatically diminished performance of $LiCoO_2$||C cells at low temperatures. A combination of poor diffusion kinetics and diminished Li ion conductivity leads to an exponential rise in charge transfer resistance (R_{CT}). At low temperatures, R_{CT} dominates cell impedance, dictating charge and discharge. Minimizing the cell's R_{CT} is critical to facilitate lithiation/delithiation. The substantially high R_{CT} of $LiCoO_2$||C cells at -40 ^oC explains the inability to charge/discharge at excessively low temperatures. Nanostructuring of Ge proved effective in lowering Li ion desolvation energy at the interface, promoting charge/discharge at such low temperatures. For capacity-matched anodes, the difference in internal resistances may arise from differences in electrode layer thicknesses and mass loadings. As graphite has a capacity approx. 4x less than germanium, a larger active mass is required to match the capacity of Ge. Additionally, the introduction of inactive binder materials further increases layer thickness.

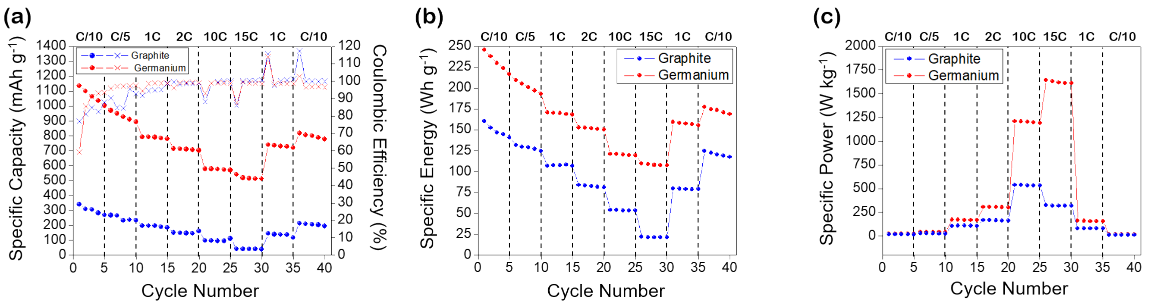

Figure S6. Comparative RCTs of $LiCoO_2$||Ge and $LiCoO_2$||C cells with a constant PN ratio of 1.5. (a) Discharge capacity and CE, (b) specific energy and (c) specific power accounting for total mass of each electrode.

Room temperature rate testing of $LiCoO_2$||Ge and $LiCoO_2$||C cells revealed superior energy and power densities of Ge NW based cells over conventional graphite-based cells (Figure S6). For accurate comparison, both cells types were matched in terms of anode areal capacity (578 µAh cm^{-2}), PN ratio (PN1.5) and voltage window. As expected, $LiCoO_2$||Ge far outperformed $LiCoO_2$||C in terms of specific capacity, reaching 548 mAh g^{-1} at 15C, compared to 45 mAh g^{-1} for $LiCoO_2$||C (Figure S6a). $LiCoO_2$||Ge far outperformed $LiCoO_2$||C in terms of specific energy and power also, reaching 109 Wh kg^{-1} and 1650 W kg^{-1} at 15C compared to 21 Wh kg^{-1} and 328 W kg^{-1} for $LiCoO_2$||C (Figure S6b-c).

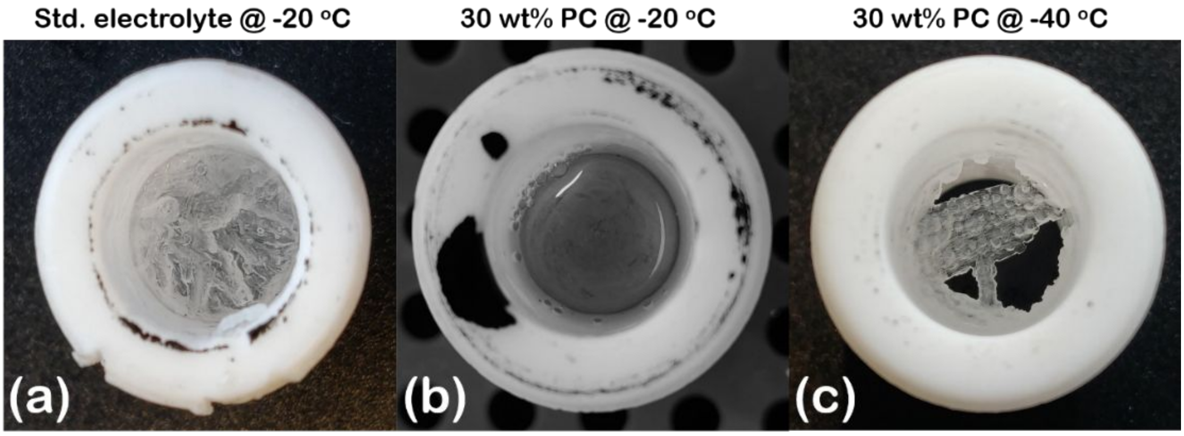

Figure S7. Images taken of the (a) std. electrolyte (1 M $LiPF_6$ in EC:DEC (1:1 v/v) + 3 wt% VC) and (b) the optimised low temperature electrolyte (1 M $LiPF_6$ in EC:DEC (1:1 v/v) + 3 wt% VC + 30 wt% PC). Cells (a) and (b) were stored at -20 °C and (c) at -40 °C for 1 week prior to imaging.

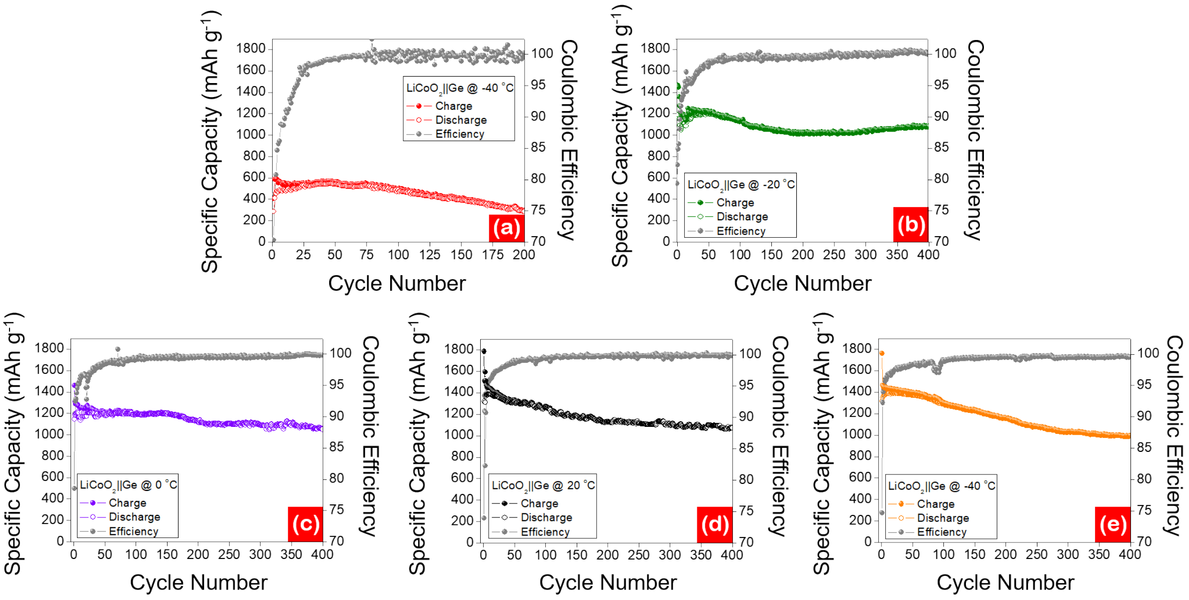

Figure S8. Charge/discharge capacities and corresponding CE of $LiCoO_2$||Ge cells at (a) -40 °C, (b) -20 °C, (c) 0 °C, (d) 20 °C and (e) 40 °C. Cells were cycled at a 1C rate between 2.8-3.9 V in temperature-specific optimised electrolytes. This data corresponds to the discharge capacity data in Figure 3d.

Charge capacities and corresponding coulombic efficiency (CE) of $LiCoO_2$||Ge cells with temperature-specific optimised electrolyte configurations (Figure S8). This data dovetails with the discharge capacity data from Figure 3d. Similar trends are noted for CE, with each cell reaching close to 100 % CE after 100 cycles. Each cell exhibited similar initial CE values of 70.44 %, 79.36 %, 78.57 %, 74.12 % and 77.98 % at -40 °C, -20 °C, 0 °C, 20 °C and 40 °C. $LiCoO_2$||Ge cells at -40 °C (Figure S8a) and -20 °C (Figure S8b) exhibit slight fluctuations in CE, attributed to Li trapping at a 1C rate.

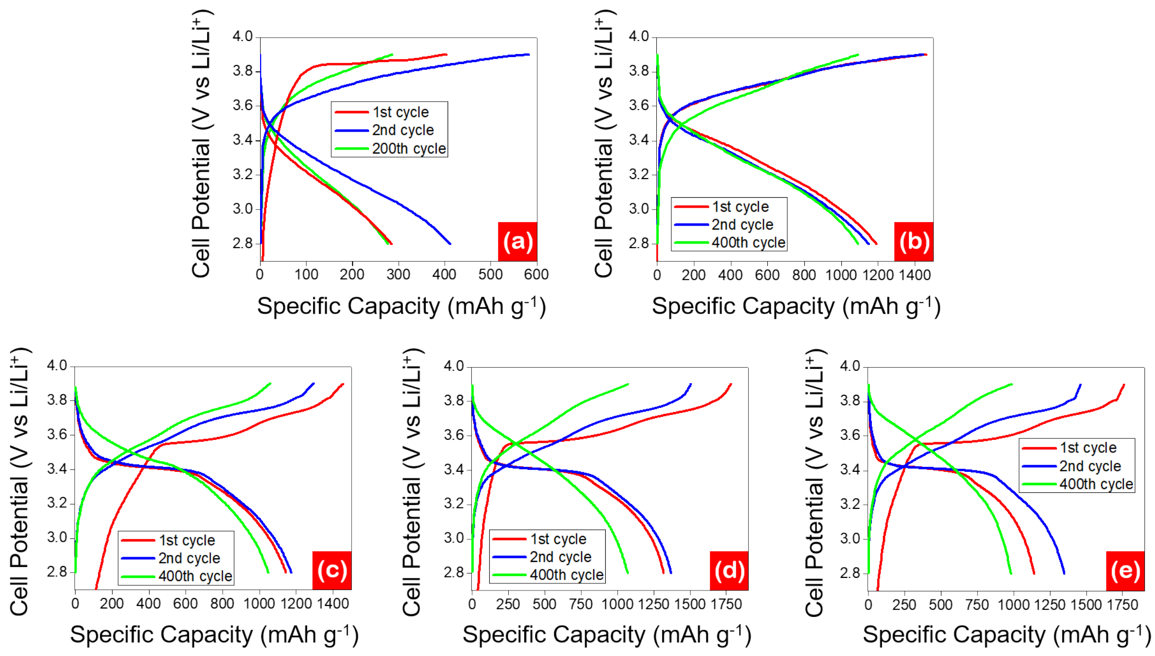

Figure S9. Capacity profiles of $LiCoO_2$||Ge cells at (a) -40 °C, (b) -20 °C, (c) 0 °C, (d) 20 °C and (e) 40 °C for the 1st (red), 2nd (blue) and final (green) cycle. Cells were cycled at a 1C rate between 2.8-3.9 V with temperature-specific optimised electrolytes. This data corresponds to the discharge capacity data in Figure 3d.

Voltage profiles of each $LiCoO_2$||Ge cell from Figure 3d are outlined in Figure S9, illustrating the 1st, 2nd and final cycle of each cell. $LiCoO_2$||Ge cells at -40 °C (Figure S9a) and -20 °C (Figure S9b) lose plateau definition, with no distinct lithiation/delithiation peaks. Instead, lithiation and delithiation seems to be a gradual capacity activation over the entire potential window. $LiCoO_2$||Ge at 0 °C (Figure S9c) is not largely affected by cell temperature, exhibiting similar profile definition to cells at 20 °C (Figure S9d) and 40 °C (Figure S9e).

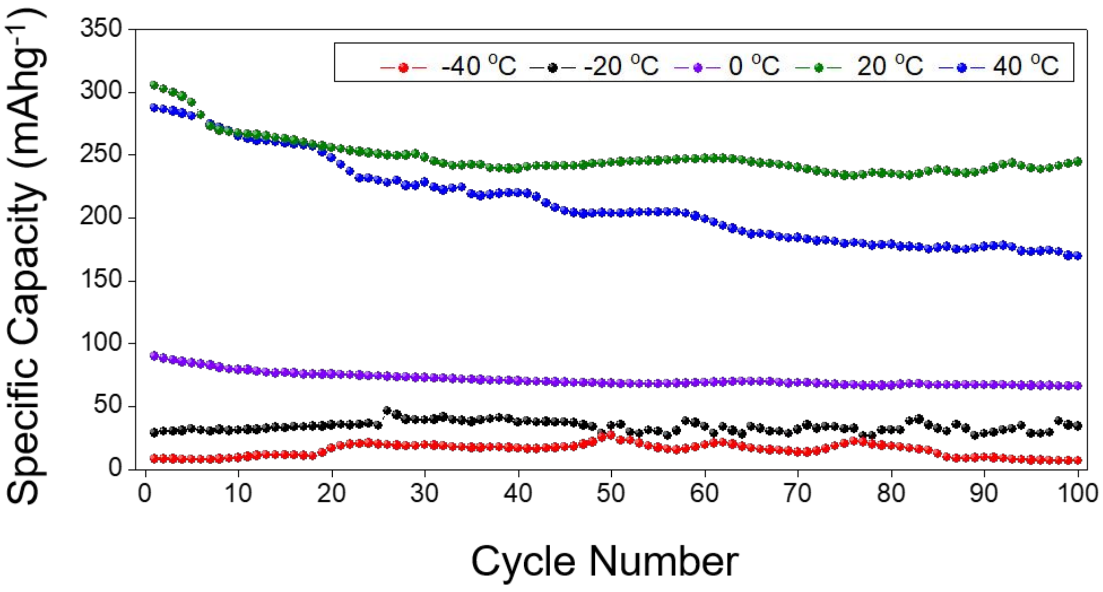

Figure S10. Discharge capacities $LiCoO_2$||C cells employing the optimised electrolyte for each temperature. From -40 ºC to 0 ºC the optimised electrolyte consists of the std. electrolyte + 30 wt% PC. At 20 ºC, the optimised RT electrolyte was taken as the std. electrolyte. At 40 ºC, the optimised electrolyte was taken as std. + EMC + 0.25 M LiBOB. Cells were cycled galvanostatically at 1C, charged and discharged symmetrically at the same temperature.

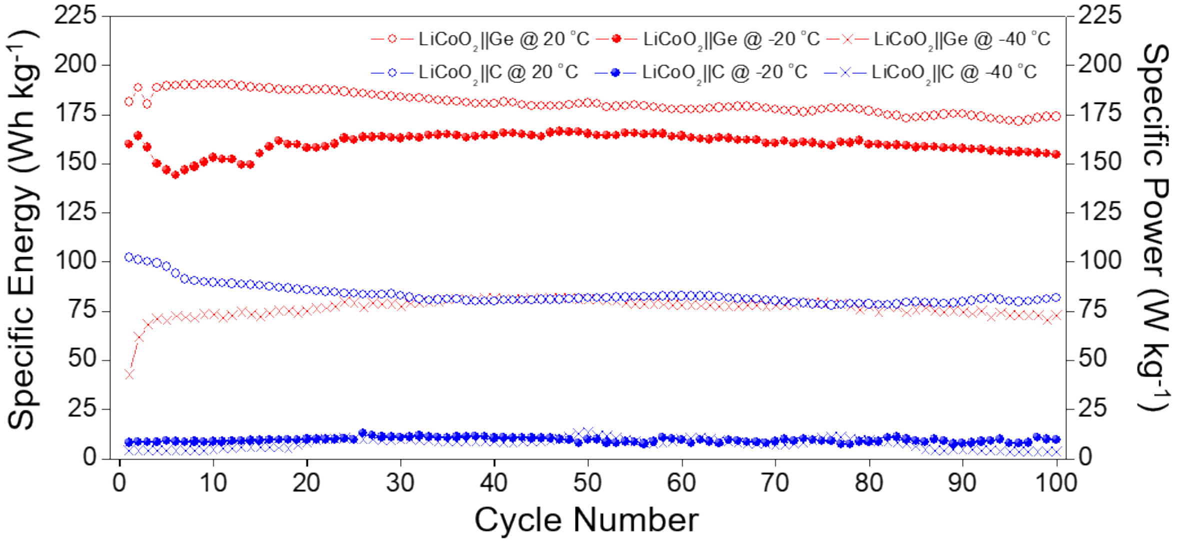

Figure S11. Specific energy and power of $LiCoO_2$||Ge and $LiCoO_2$||C cells at a 1C rate. Values for $LiCoO_2$||Ge and $LiCoO_2$||C cells were calculated from the discharge capacity values from Figure 3d and Figure S10, respectively. Values were calculated taking total electrode masses into consideration.

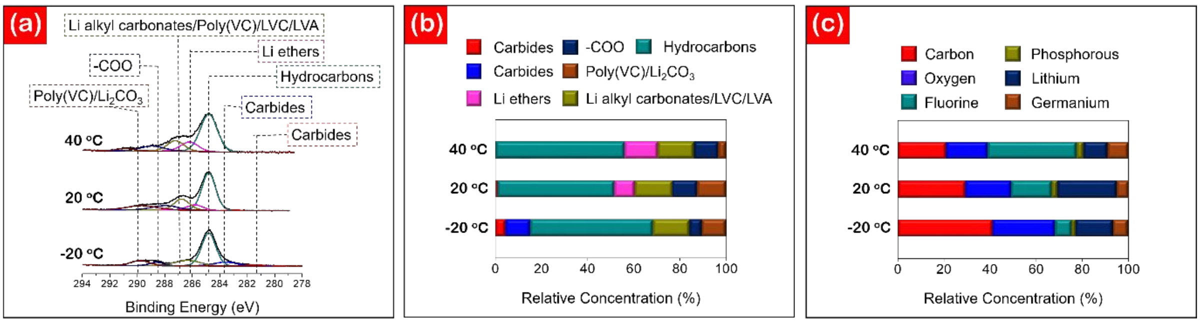

Figure S12. XPS analysis of the SEI layer after 25 cycles at -20 °C, 20 °C and 40 °C with the standard electrolyte. (a) High resolution C1s spectra, (b) relative concentrations from the C1s spectra and (c) relative elemental concentrations taken from the survey scan.

XPS was used to track variations in SEI composition as a function of temperature (Figure S12a-c). Three $LiCoO_2$||Ge cells were cycled for 25 cycles at low T, RT and high T using the std. electrolyte solution. From (a-b) the most notable differences are the presence of carbide species and the absence of Li ethers at low T. Low temperatures suppress Li ether incorporation with the Li ether composition scaling with temperature (0 % at -20 °C, 8.8 % at 20 °C and 14.3 % at 40 °C). Lithium carbide species appear only at low temperatures, suggesting a temperature dependent formation. From the literature, carbides are formed from lithium decomposition reactions whereby plated lithium decomposes to form carbide and carbonate species.[7] Elemental concentrations were collated from the survey scans (Figure S12c). The fluorine content of the SEI layer scales proportionally with temperature, indicating a greater production of fluorine-containing species at elevated temperatures. Low

temperatures suppress fluoride incorporation into the SEI, with fluorine accounting for 7.2 %, 17.7 % and 38.6 % at -20 ^{o}C, 20 ^{o}C and 40 ^{o}C, respectively.

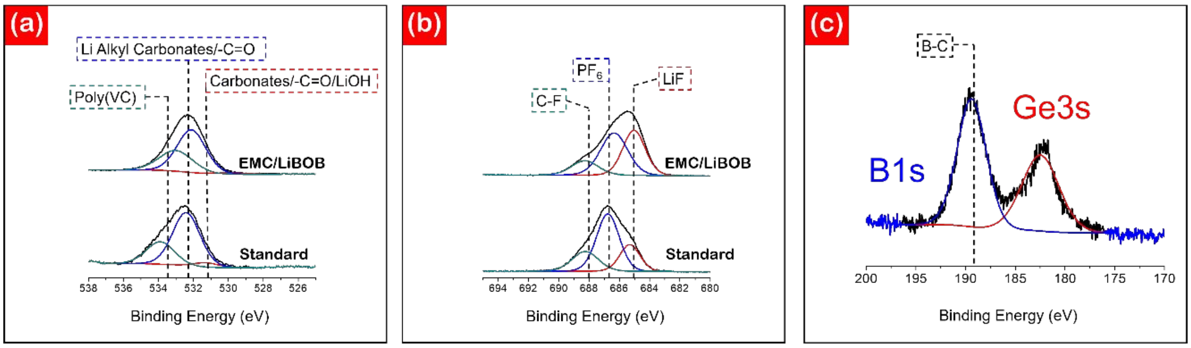

Figure S13. High resolution (a) O1s, (b) F1s and (c) B1s/Ge3s spectra of Ge NWs cycled for 25 cycles at 40 ^{o}C with the standard electrolyte and the optimised high temperature electrolyte. The data dovetails with the data illustrated in Figure 4a-c.

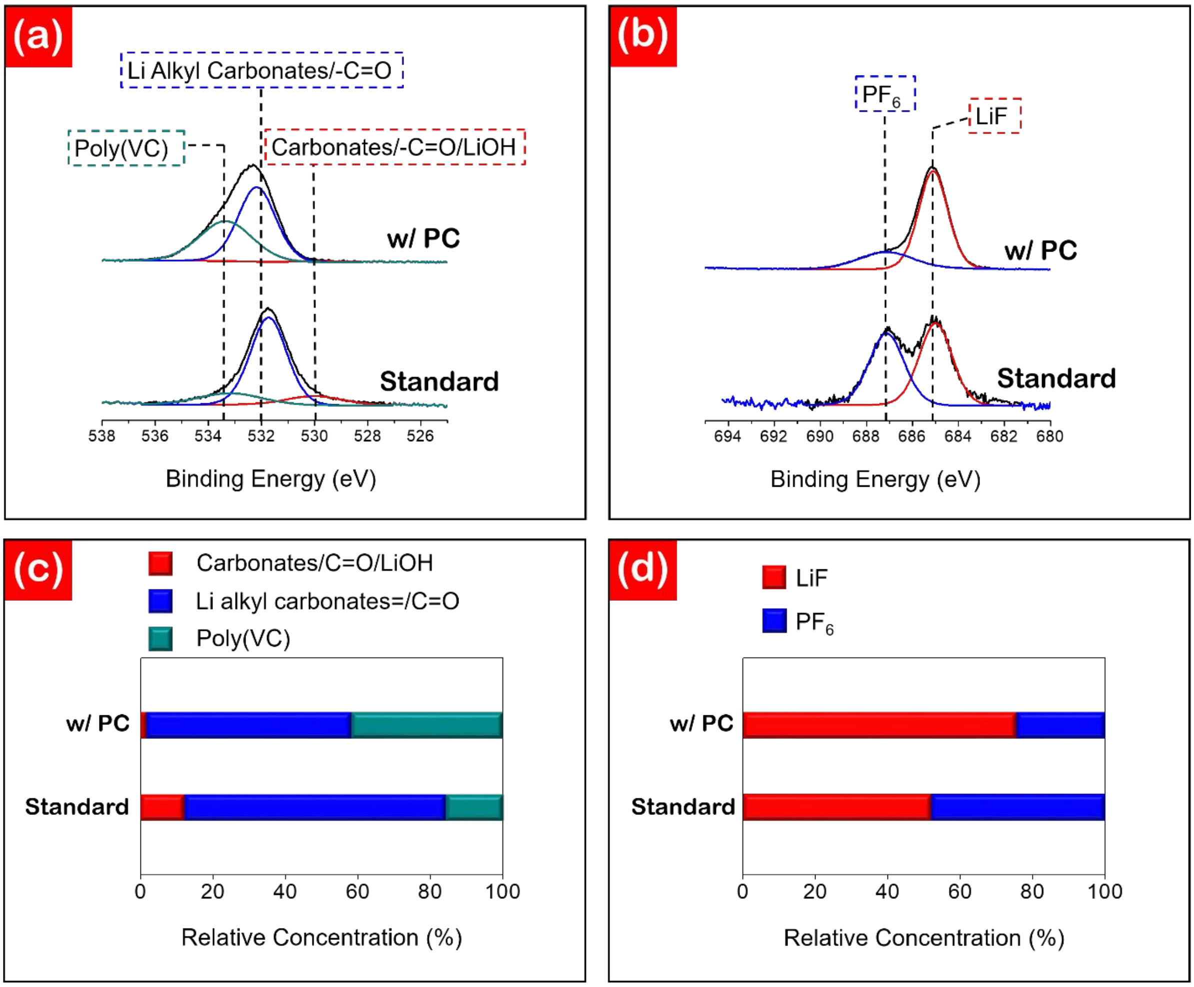

Figure S14. High resolution (a) O1s and (b) F1s spectra of Ge NWs cycled for 25 cycles at -20 ^{o}C with the standard electrolyte and the optimised low temperature electrolyte. Relative concentrations of the chemical species of the SEI layer acquired from the (c) O1s and (d) F1s spectra. The figures complement the data illustrated in Figure 4d-f.

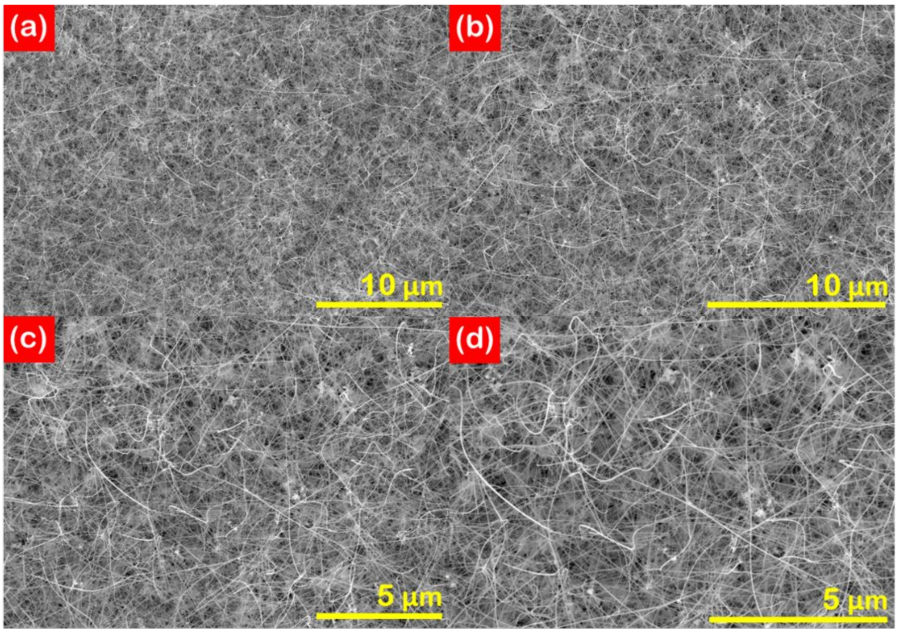

Figure S15. SEM analysis of pristine Ge NWs at different magnifications. A combination of Cu_3Ge-seeded and self-seeded Ge NWs is apparent.

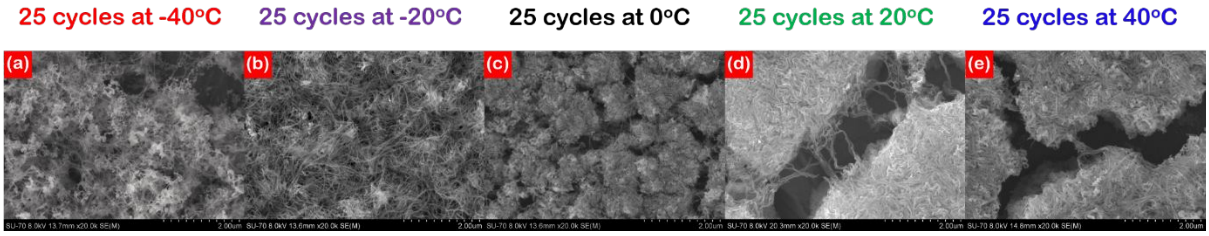

Figure S16. SEM images of Ge NW anodes cycled for 25 cycles at (a) -40 °C, (b) -20 °C, (c) 0 °C, (d) 20 °C and (e) 40 °C. Each cell was cycled using the corresponding optimised electrolyte for that temperature (Figure 2). Note that (d) matches Figure 5(i) as the std. electrolyte is taken as the optimized RT electrolyte.

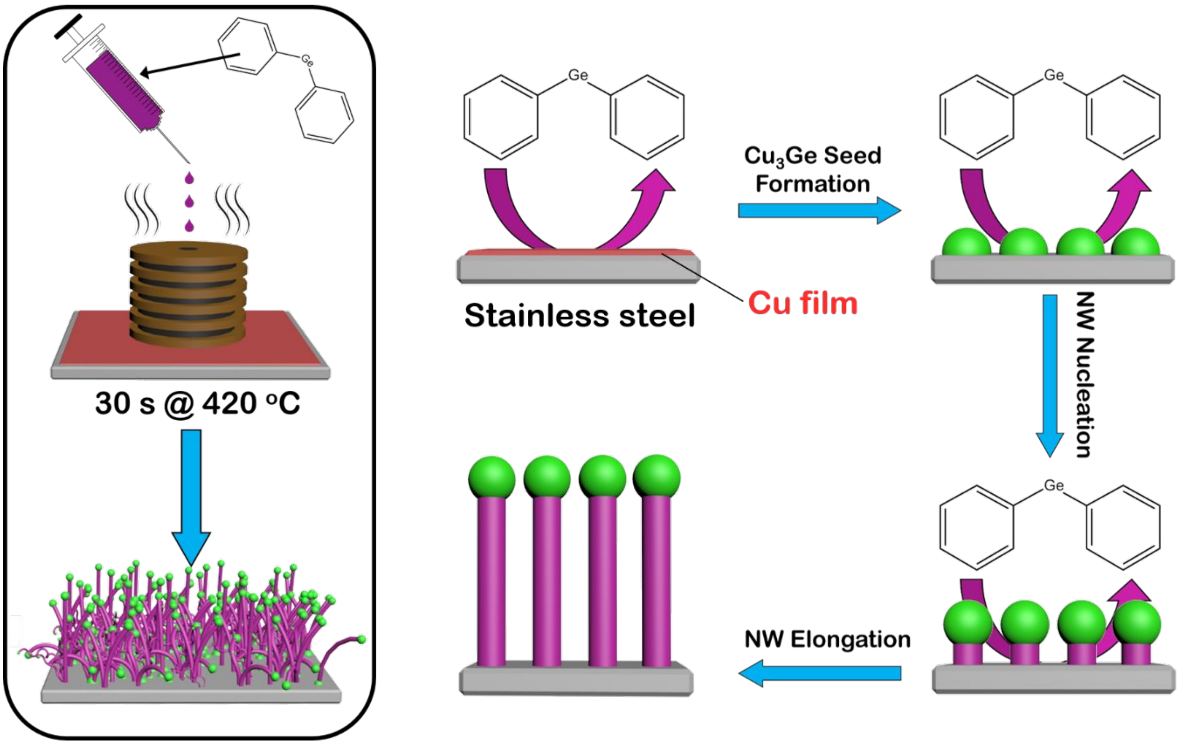

Figure S17. Schematic illustration of the rapid solvent-free method employed in synthesizing germanium nanowires. The reaction is driven by a vapor-solid-solid (VSS) mechanism. High temperature decomposition of diphenylgermane promotes surficial adsorption of Ge monomers on the Cu-coated SS substrate, Cu_3Ge seed formation and subsequent NW nucleation.

REFERENCES

1. Choi, S.; Cho, Y. G.; Kim, J.; Choi, N. S.; Song, H. K.; Wang, G.; Park, S., Mesoporous Germanium Anode Materials for Lithium-Ion Battery with Exceptional Cycling Stability in Wide Temperature Range. *Small* **2017,** *13* (13), 1603045.

2. Markevich, E.; Salitra, G.; Aurbach, D., Low temperature performance of amorphous monolithic silicon anodes: comparative study of silicon and graphite electrodes. *Journal of The Electrochemical Society* **2016,** *163* (10), A2407.

3. Ma, W.; Wang, Y.; Yang, Y.; Wang, X.; Yuan, Z.; Liu, X.; Ding, Y., Temperature-Dependent Li Storage Performance in Nanoporous Cu–Ge–Al Alloy. *ACS applied materials & interfaces* **2019,** *11* (9), 9073-9082.

4. Varzi, A.; Mattarozzi, L.; Cattarin, S.; Guerriero, P.; Passerini, S., 3D Porous Cu–Zn Alloys as Alternative Anode Materials for Li-Ion Batteries with Superior Low T Performance. *Advanced Energy Materials* **2018,** *8* (1), 1701706.

5. Yan, Y.; Ben, L.; Zhan, Y.; Huang, X., Nano-Sn embedded in expanded graphite as anode for lithium ion batteries with improved low temperature electrochemical performance. *Electrochimica Acta* **2016,** *187*, 186-192.

6. Elia, G. A.; Nobili, F.; Tossici, R.; Marassi, R.; Savoini, A.; Panero, S.; Hassoun, J., Nanostructured tin–carbon/LiNi0. 5Mn1. 5O4 lithium-ion battery operating at low temperature. *Journal of Power Sources* **2015,** *275*, 227-233.

7. Ng, B.; Coman, P. T.; Faegh, E.; Peng, X.; Karakalos, S. G.; Jin, X.; Mustain, W. E.; White, R. E., Low-Temperature Lithium Plating/Corrosion Hazard in Lithium-Ion Batteries: Electrode Rippling, Variable States of Charge, and Thermal and Nonthermal Runaway. *ACS Applied Energy Materials* **2020,** *3* (4), 3653-3664.
